# Hit-to-Lead Studies of Pyrazinylpiperazines against Visceral Leishmaniasis: Pharmacokinetic Profile and *In Vivo* Efficacy of Potent Compounds against *Leishmania infantum*

**DOI:** 10.1021/acsptsci.5c00318

**Published:** 2025-07-18

**Authors:** Thibault Joseph William Jacques Dit Lapierre, Mariza Gabriela Faleiro de Moura Lodi Cruz, Gisele Barbosa, Analu R. Costa, Miguel Angel Chávez-Fumagalli, Thamires Quadros Froes, Priscila Zonzini Ramos, Paula Derksen Macruz, Thalita Carolyne Souza Trindade, Eduardo Jorge Pilau, Patricia Sampaio Tavares Veras, Maria Cristina Nonato, Katlin B. Massirer, Leonardo L. G. Ferreira, Adriano D. Andricopulo, Lídia Moreira Lima, Silvane Maria Fonseca Murta, Celso de Oliveira Rezende Júnior

**Affiliations:** a Laboratório de Síntese de Candidatos a Fármacos, Instituto de Química, 28119 Universidade Federal de Uberlândia (UFU), Uberlândia, MG 38400-902, Brazil; b Grupo de Genômica Funcional de Parasitos, Instituto René Rachou, 154611 Fundação Oswaldo Cruz (FIOCRUZ Minas), Belo Horizonte, MG 30190-002, Brazil; c Laboratório de Avaliação e Síntese de Substâncias Bioativas (LASSBio ®), 206381 Universidade Federal do Rio de Janeiro, Rio de Janeiro, RJ 21941-902, Brazil; d Laboratório de Química Medicinal e Computacional (LQMC), Instituto de Física de São Carlos (IFSC), Universidade de São Paulo (USP), São Carlos, SP 13563-120, Brazil; e Computational Biology and Chemistry Research Group, Vicerrectorado de Investigación, 187079 Universidad Católica de Santa María, Arequipa 04000, Peru; f Laboratory of Host-Parasite Interaction and Epidemiology Gonçalo Moniz Institute - Fiocruz − Bahia, Salvador, BA 40296-710, Brazil; g Center for the Research and Advancement in Fragments and molecular Targets (CRAFT), School of Pharmaceutical Sciences at Ribeirao Preto, 67782 University of São Paulo, Ribeirão Preto, SP 14040-903, Brazil; h Protein Crystallography Laboratory, Department of Biomolecular Sciences, School of Pharmaceutical Sciences at Ribeirao Preto, University of São Paulo, Ribeirão Preto, SP 14040-903, Brazil; i Centro de Química Medicinal (CQMED), Centro de Biologia Molecular e Engenharia Genética (CBMEG), 28132 Universidade Estadual de Campinas (UNICAMP), Campinas, SP 13083-886, Brazil; j Laboratório de Biomoléculas e Espectrometria de Massas (LaBioMass), 42487 Universidade Estadual de Maringá (UEM), Maringá, PR 807020-900, Brazil

**Keywords:** pyrazinylpiperazines, visceral leishmaniasis, lead discovery, pharmacokinetics, mechanism of action

## Abstract

As part of the hit-to-lead optimization of antileishmanial pyrazinylpiperazines, the Absorption, Distribution, Metabolism, and Excretion (ADME) properties of the initial hit and five candidates for *in vivo* studies were assessed *in vitro*. These candidates, which displayed improved potency against *Leishmania infantum* (IC_50_ < 5 μM), were selected from an earlier structure–activity relationship study. Such assessment revealed that, except for the catechol derivative **6**, all derivatives exhibited an improved overall ADME profile in comparison to the initial hit. The *para*-hydroxyl analog **2** stood out as the most promising candidate, being the second most potent compound *in vitro* against the parasite and showing far superior metabolic stability (more than twice as stable as the initial hit) in mouse liver microsomes, together with a reasonable gastrointestinal absorption and a lack of blood–brain barrier permeation. *In vivo* assessment of the antileishmanial efficacy of **2** in a BALB/c mice model of visceral leishmaniasis revealed a reduction in parasitemia by 91.1 and 90.0% in the spleen and liver, respectively, after a 10 day treatment of infected animals with a 100 mg/kg daily dose of **2**, without any acute toxicity or death among mice treated with **2**. Flow cytometry demonstrated that the antileishmanial activity of **2** is linked to a cytostatic effect, marked by cell cycle arrest in the G0/G1 phase and enhanced production of reactive oxygen species. Subsequent *in silico* studies suggested that the activity of the novel antileishmanial pyrazinylpiperazine lead **2** could be due to the inhibition of a nonspecific serine/threonine protein kinase in *Leishmania infantum*; however, *in vitro* inhibition assays failed to identify a target for **2** among a set of kinases and other proteins.

## Graphical abstract

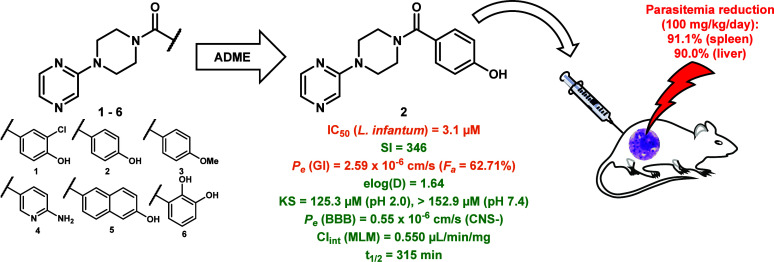

Visceral leishmaniasis (VL) is a neglected tropical disease (NTD) that affects 50,000 to 90,000 people and is responsible for 20,000 to 40,000 deaths each year.^1–3^ It is spread worldwide, with Southeastern Asia and Southern America being the most affected regions and six countries (Brazil, India, Nepal, Bangladesh, Sudan, and Ethiopia) accounting for 90% of newly reported cases.^3,4^

VL is caused by either *Leishmania donovani* (*L. donovani*) or *Leishmania infantum* (*L. infantum*), two protozoan parasites from the *Leishmania* genus, depending mainly on the geographical location, although other *Leishmania* species were linked to VL cases, with a considerably lesser influence than *L. donovani* and *L. infantum* nonetheless.^5^ Transmission occurs via an insect vector, namely *Phlebotominae* spp. sandflies, the bite of which releases the parasite in its promastigote morphological form in the skin.^5,6^ Within the mononuclear phagocytic system of the vertebrate host, the protozoan differentiates into its amastigote form and infects various cells and tissues of the vertebrate host, including macrophages. The transmission cycle of the disease is completed when blood containing *Leishmania*-infected macrophages is ingested by a sandfly when biting the human host, thus transmitting the parasite back to the insect vector, where the amastigotes can differentiate once more into promastigotes and migrate to the proboscis, enabling further transmission of the disease by the newly infected sandfly.^6,7^

Common clinical manifestations of human VL include prolonged fevers, body weight loss, anemia, pancytopenia, skin darkening, splenomegaly, and hepatomegaly, the latter two being the consequence of a migration of the parasite to the spleen and liver, respectively.^8,9^ It is crucial to ensure that the disease is promptly treated, as the lethality of untreated VL is as high as 90%.^6,8^

However, despite the critical need to address new cases of VL, the therapeutic options for such treatment remain scarce. Amphotericin B (available in diverse formulations), miltefosine, sodium stibogluconate, pentamidine and meglumine antimoniate (Glucantime) (Figure 1) are among the few antileishmanial treatments approved by regulatory agencies across the world.^10,11^ Nonetheless, such compounds are plagued by a wide array of shortcomings, including their high cost per dose, high toxicity, and the rise of resistant strains in some regions.^11–13^

**Figure 1:**
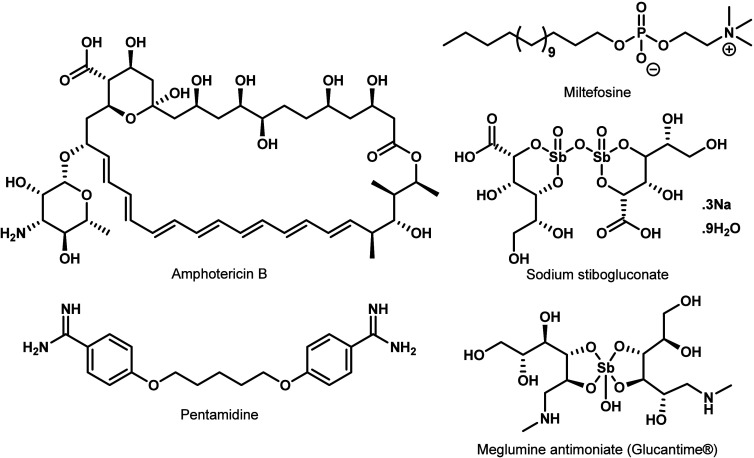
Compounds used for the treatment of VL.

Given the current situation of VL and its treatment, a rising number of hit-to-lead and lead optimization campaigns have been performed toward the goal of identifying new, more efficient, safer, and ideally more affordable drug candidates against VL.^14–18^ Among these recent efforts, our team recently published a structure–activity relationship (SAR) of the pyrazinylpiperazine scaffold, in which 40 benzoyl analogs of the initial hit **1** (Figure 2) were assayed against *L. infantum* and *L. braziliensis*, as part of the hit-to-lead optimization of this class of compounds.^19^ The hit itself was selected from a GSK chemical library, known as LeishBox, which regroups compounds identified as antileishmanial hits by high-throughput screening;^20^ and the SAR identified eight lead candidates, highly potent (IC_50_ ≤ 5 μM) against *L. infantum* with low cytotoxicity toward host cells (SI ≥ 184).^19^

**Figure 2:**
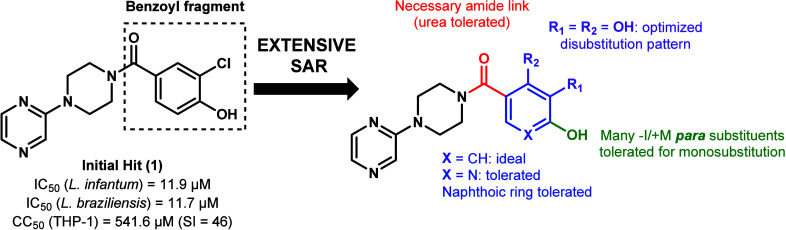
Structure and biological properties of **1**, and graphical summary of the SAR previously reported.

Herein, we report the assessment of the ADME profile *in vitro* of five lead candidates selected from the previous study, as part of the hit-to-lead process for *in vivo* candidates, as well as the *in vivo* efficacy of the *para*-hydroxyl derivative **2** in a VL mice model. *In silico* investigation of the mechanism of action (MoA) of **2** is also reported.

## Results and Discussion

### Chemistry

All potential lead candidates were synthesized as described in a previous publication.^19^ Briefly, the pyrazinylpiperazine core of these compounds was achieved by submitting 2-chloropyrazine to a nucleophilic aromatic substitution with an excess of piperazine, yielding the amine **8**, which subsequently underwent EDC/HOBt-mediated amide coupling with the appropriate carboxylic acids to afford the target compounds **1** – **5** and **7** (Scheme 1). Finally, the 2,3-dimethoxy intermediate **7** required an additional demethylation using BBr_3_ as demethylating agent to provide the catechol **6** (Scheme 1).

**Scheme 1:**
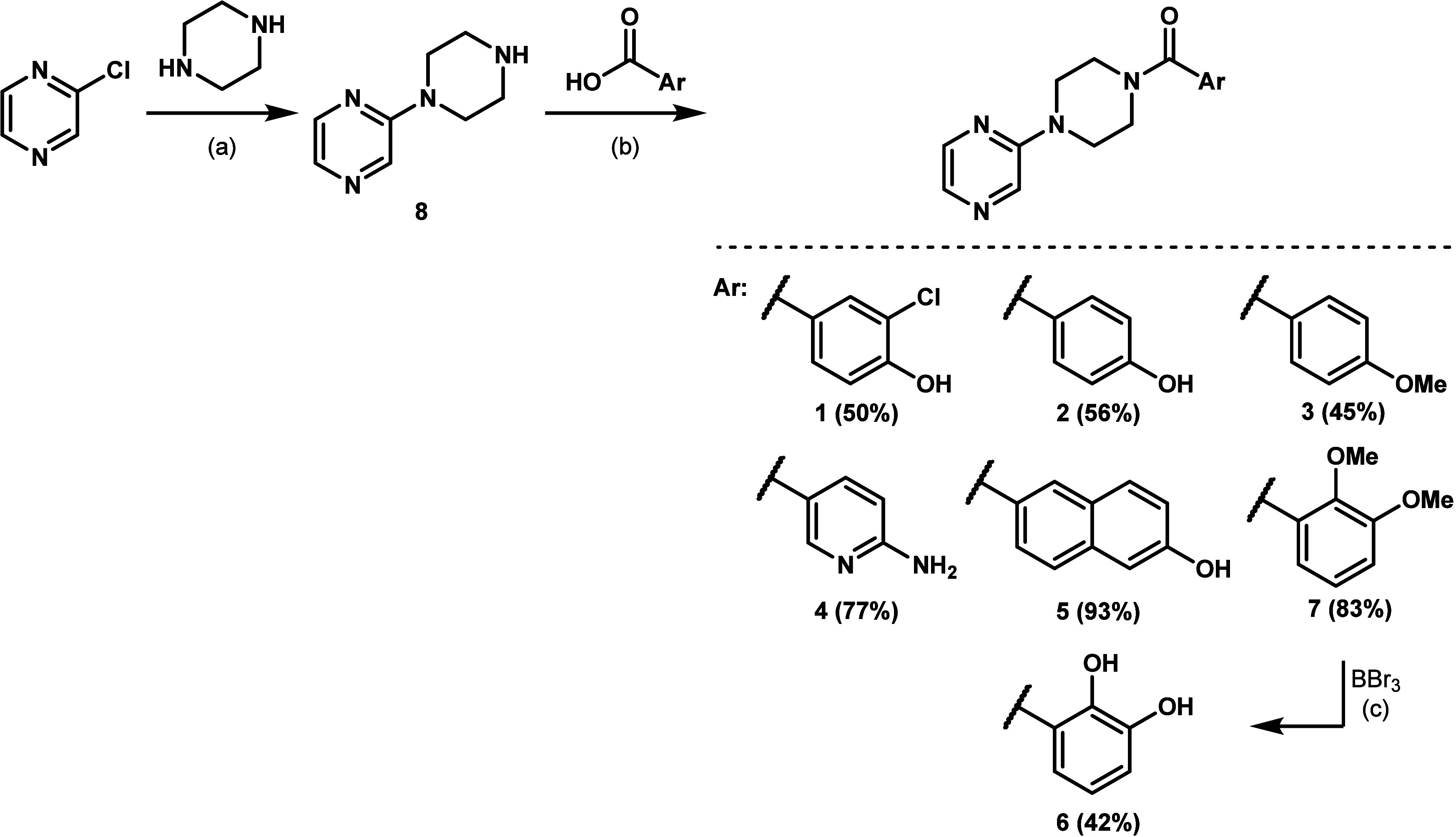
Synthesis of Hit **1** and Potential Lead Candidates **2** – **6** ^a^

a Reagents and conditions: (a) Na_2_CO_3_, iPrOH, 80 °C, 24 h, 100%; (b) EDC, HOBt, DMF, rt, 40 – 72 h, 45 – 93%; (c) BBr_3_ (1 M in DCM), DCM, Ar atm., 0 °C – rt, 21 h, 42%.

Quite noteworthy, when scaling up the synthesis of **2** from **8** and 4-hydroxybenzoic acid, the yield dropped by approximately 20%, from 56% on a 20 mg scale (as previously reported) to 37% on a 400 mg scale. Therefore, an alternative route has been considered for the synthesis of **2** on larger scales, revolving around the use of an acyl chloride for the amide coupling between **8** and the carboxylic acid, considering the higher reactivity of acyl chlorides when compared to EDC/HOBt activated esters in amide couplings (Scheme 2). Initially, a simple amide coupling via acyl chloride formation between *para*-hydroxybenzoic acid and **8** was attempted, using thionyl chloride to generate the acyl chloride, but proved unsuccessful, probably due to side reactions involving the *para*-hydroxyl group of the carboxylic acid (hydroxy-chlorine exchange and/or esterification, mainly). Thus, *para*-hydroxybenzoic acid was first protected with an acetyl group to prevent the aforementioned side reactions in the next step, yielding *para*-acetoxybenzoic acid (**9**) quantitatively. Next, amide coupling between **8** and **9** was achieved in a satisfying 67% yield on a 170 mg scale, via formation of an acyl chloride intermediate from **9** using oxalyl chloride. Finally, the acetyl protection was removed by hydrolysis in basic conditions, yielding **2** in 99% yield from **10** and in 66% yield over three steps from *para*-hydroxybenzoic acid, greatly improved from the moderate yield of 37% obtained with EDC/HOBt-mediated amide coupling.

**Scheme 2:**
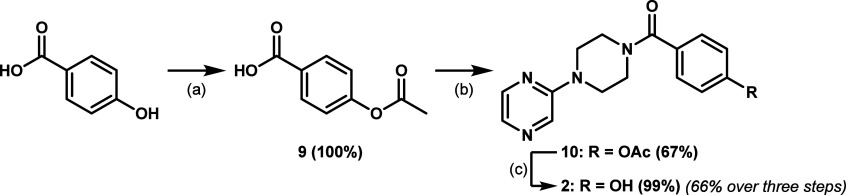
Optimized Three-Step Synthesis of **2** on a Larger Scale^a^

a Reagents and conditions: (a) Acetic anhydride, H_2_SO_4_ (96%) (cat.), 80 °C, 16 h, 100%; (b) (COCl)_2_, DMF (cat.), DCM, Ar atm., 0 °C, 2 h *then* **8**, Et_3_N, DCM, Ar atm., 0 °C – rt, 3 h, 67%; (c) NaOH (2 M), MeOH/THF (3:1), rt, 3 h, 99%.

### *In Vitro* Assessment of Hit 1 and Lead Candidates 2 – 6

#### ADME Profile of Hit **1** and Lead Candidates **2** – **6**

To identify the most suitable compound for *in vivo* assessment in mice models, the ADME profile of the initial hit **1** and the benzoyl derivatives **2** – **6** from the previous SAR study which exhibited the highest potency (IC_50_ < 5 μM) against *L. infantum* amastigotes was determined *in vitro*. Some compounds from the SAR analysis meeting this potency criterion were deliberately not considered for the present study, as they featured either PAINS fragments or labile groups, such as a *N*-Boc protecting group which upon acidic cleavage would yield a poorly active amine (IC_50_ increasing from 2.9 μM to 86.5 μM). Nonetheless, **6** was selected for *in vitro* ADME evaluation given its activity against both *L. infantum* and *L. braziliensis* and low cytotoxicity against host cells, despite its catechol moiety being considered as PAINS.^21^

The set of ADME properties assessed *in vitro* for *in vivo* candidates **1** – **6** was selected to provide an accurate estimate of both their bioavailability and metabolic stability. Thus, the blood-brain barrier (BBB) and gastrointestinal (GI) permeability were assessed by Parallel Artificial Membrane Permeability Assay (PAMPA). As VL mainly affects internal organs such as the spleen and liver, rather than the brain or the central nervous system, a satisfactory GI permeability combined with low BBB permeability would suggest that compounds should readily reach organs with a high parasite burden without exerting any side-effects associated with interactions with the central nervous system. The microsomal half-life and intrinsic clearance of all compounds were also determined in Mouse Liver Microsomes (MLM), in the presence of a NADPH-generating system. The resulting *in vitro* ADME profiles of **1** – **6** are summarized in Table 1.

**Table 1:**
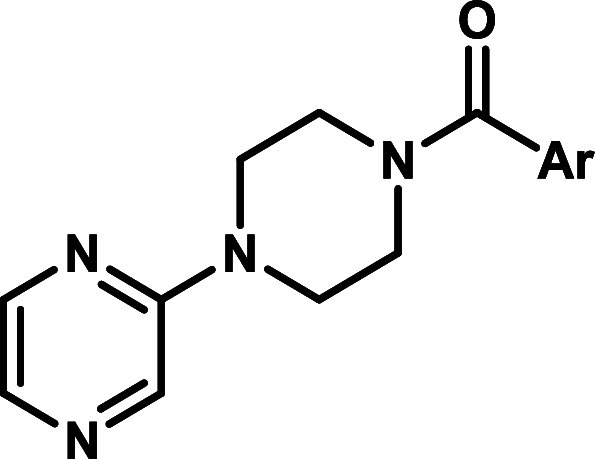

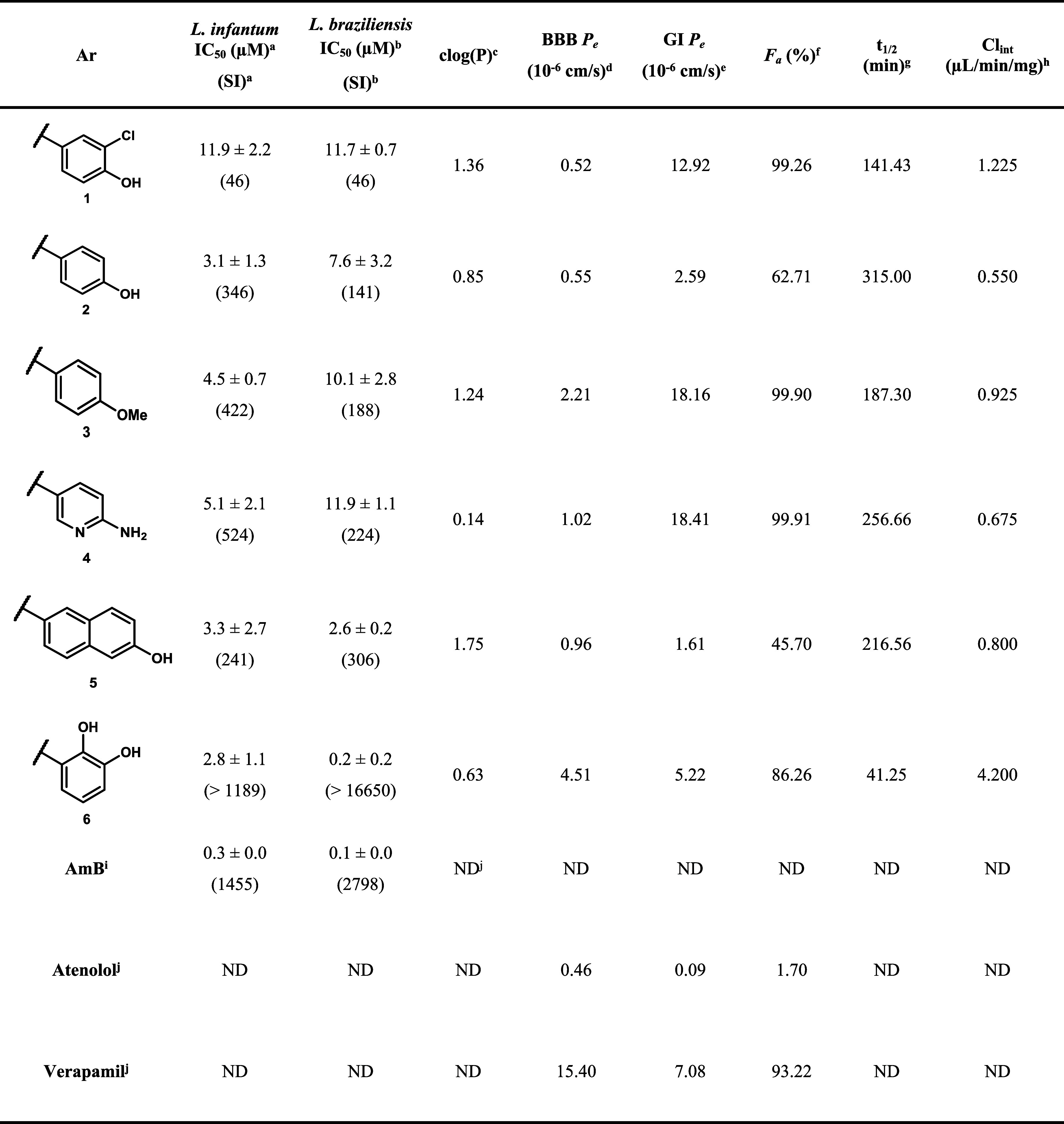
*In Vitro* Antileishmanial Potency, Selectivity Index, and ADME Properties of Compounds **1** – **6**

a IC_50_ value (μM) for the inhibition of the growth of *L. infantum* in THP-1 macrophages (mean of at least two independent determinations, *P* < 0.05), SI: selectivity index.

b IC_50_ value (μM) for the inhibition of the growth of *L. braziliensis* in THP-1 macrophages (mean of at least two independent determinations, *P* < 0.05), SI: selectivity index.

c log­(P) values were calculated using SwissADME (http://www.swissadme.ch/index.php).^22^

d Blood-brain barrier permeability. CNS-: low BBB permeation, *P*_*e*_ (10^–6^ cm/s) **<** 2.0. CNS±: uncertain BBB permeation, 2.0 < *P*_*e*_ (10^–6^ cm/s) **<** 4.0. CNS+: high BBB permeation, *P*_*e*_ (10^–6^ cm/s) **>** 4.0.^23,24^

e Gastrointestinal permeability.

f Fraction absorbed (GI). High absorption: *F*_*a*_ > 70%, intermediate absorption: 30% < *F*_*a*_ < 70%, low absorption: *F*_*a*_ < 30%.^25^

g Microsomal half-life determined in Mouse Liver Microsomes (MLM), in the presence of NADPH cofactor.

h Intrinsic clearance determined in MLM, in the presence of NADPH cofactor.

i Amphotericin B (reference drug) was used as positive control for *in vitro* antileishmanial assays.

j Atenolol and Verapamil were used as positive and respective controls, respectively, for BBB and GI permeability assays; ND: Not determined.

Overall, the initial hit **1** and all lead candidates **2** – **6** showed satisfactory ADME profiles for *in vivo* evaluation.^26–28^ Except **3** and **6**, all compounds exhibited a low BBB permeability and were thus classified as CNS- (Table 1). The 4-methoxyphenyl analog **3** showed a permeability value in the uncertainty range of the assay (CNS±), preventing it from being clearly classified as CNS positive (CNS+) or negative (CNS-), and **6** was found to readily permeate the BBB (CNS+). Therefore, it can be speculated that compounds **1**, **2**, **4** and **5** could display limited off-target interactions with the central nervous system and other possible side-effects associated with BBB permeation. Regarding GI absorption, the initial hit already proved to readily permeate the GI tract, displaying high *P*_*e*_ and *F*_*a*_ values (**1**, Table 1), and both the *para*-methoxybenzoic and 6-aminonicotinic derivatives managed to further improve it, when compared to **1** (compounds **3** and **4**, Table 1). Quite surprisingly, **4** achieved such a feat despite exerting the lowest lipophilicity of the set of lead candidates (clog­(P) = 0.14, Table 1), even exhibiting the highest GI absorption of all derivatives considered in this work. In contrast, the chlorine removal in **2** and the replacement of the 3-chloro-4-hydroxybenzoic ring by a 2-naphthol moiety in **5** led to rather average GI absorption for these two compounds, the latter even exhibiting *F*_*a*_ < 50%. Such a decrease in permeability, when compared to **1**, could be expected and explained by a lower lipophilicity for **2**, but not in the case of the naphthoic derivative **5**, which displayed the highest clog­(P) of all lead candidates. Instead, the lower GI permeability of **5** might be due to the sole presence of its *para*-hydroxyl group, which can also contribute to explaining the lower GI permeability of **2** in addition to the lipophilicity-based explanation previously provided. The *para*-hydroxyl substituent being a sterically unhindered hydrogen bond acceptor and donor, it therefore contributes to decreasing not only the lipophilicity of **2** and **5**, when compared to their 3-chloro-4-hydroxyl equivalents, but also their ability to cross cellular membranes, by allowing hydrogen bonding with said membranes more easily without the steric restrictions exerted by the neighboring chlorine. Different from the two monohydroxylated derivatives, the catechol **6** exhibited a somewhat higher GI absorption (*F*_*a*_ = 86.26%, Table 1) despite its additional hydroxy group and somewhat lower lipophilicity (clog­(P) = 0.63). Such higher GI absorption, when compared to **2** and **5**, could be due to intramolecular hydrogen bonding on the catechol fragment of **6**. Indeed, intramolecular hydrogen bonds can significantly contribute to increasing the membrane permeability of small molecules, by mimicking the removal of one H-bond acceptor and one H-bond donor and thus “shielding” the polarity of the molecule, resulting in increased permeability.^29–31^ Therefore, the GI permeability and absorption of pyrazinylpiperazines might not exclusively depend on the nature of the substituents featured on their benzoyl ring, but also on their number and relative positions, as exemplified by the comparison between **2**, **5** and **6**. Satisfyingly, almost all lead candidates for *in vivo* assessment displayed improved metabolic stability, when compared to **1**. The only exception was the catechol derivative **6**, for which the intrinsic clearance was three times higher than the initial hit and the microsomal half-life decreased by the same factor. Such results are to be expected with catechol-containing compounds such as **6**, as this fragment is known for being prone to oxidative metabolism by CYP enzymes into highly reactive quinoid metabolites and therefore classified as PAINS.^21,32^ This, in addition to its CNS+ liability, led **6** to be ruled out of the potential candidates for *in vivo* assessment against *L. infantum*, in spite of its superior potency and low cytotoxicity *in vitro*. The *meta*-Cl removal in **2** proved the most efficient at increasing metabolic stability, as the intrinsic clearance of **2** was more than halved, and its microsomal half-life 2.2 times higher, when compared to **1**, which could be expected given the substantial reduction in lipophilicity from **1** to **2** (Table 1). Interestingly, the methoxy counterpart of **2** (compound **3**) only reduced the clearance by a factor 1.3 when compared to **1**, whereas the other close relative of **2**, the 6-aminonicotinic derivative **4**, showed similar results to **2** in terms of metabolic stability. A possible explanation for such different metabolic profiles among such similar compounds could be that methoxylated derivatives can be readily metabolized through demethylation, suggesting that the methoxy group of **3** is responsible for its lower metabolic stability than its closely related analogs **2** and **4**.^33^ Nonetheless, and despite the lower microsomal stability of **3** when compared to **2**, *in vivo* metabolism of **3** is very likely to mainly yield **2** via demethylation, effectively releasing a more active compound as the main metabolite.

Considering all the aforementioned results, the *para*-hydroxy derivative **2** was identified as the most promising lead candidate, displaying the best overall ADME profile and one of the highest antileishmanial potencies. Despite its relatively lower GI permeability and absorption than **4**, **2** remained with the lowest BBB permeability and the highest microsomal metabolic stability among the lead candidates, which might counterbalance the lower GI absorption to ensure a satisfactory bioavailability. Additionally, **2** exhibited adequate lipophilicity (elog­(D) = 1.64), though no correlation could be observed between lipophilicity and antileishmanial activity among the set of lead candidates, satisfactory water solubility (kinetic solubility = 125.3 μM (pH 2.0) and >152.9 μM (pH 7.4)) and low cytotoxicity against HepG2 cells (CC_50_ > 64 μM), suggesting that it should not exert hepatotoxicity. Therefore, **2** was deemed suitable for *in vivo* assessment and was selected for the assessment of its antileishmanial efficacy and toxicity in mice models, and complementary *in vitro* and *in silico* efforts aimed at identifying the mode of action and molecular target of **2** were performed.

#### Synergism between **2** and Antimonial Drugs

Synergism is characterized by the combination of two drugs resulting in a greater effect than the one expected based on the simple additivity of their individual activities.^34^ One strategy to tackle the emergence of drug-resistant strains of *Leishmania* parasites is the use of combination therapy, which relies, at least partially, on the synergistic effects of two drugs with distinct mechanisms of action.^1^ Therefore, assessing possible synergistic effects during the development of novel antileishmanial candidates is a relevant step of the process, regarding combination therapy.^1,13^

Pentavalent antimonials are supposedly acting as prodrugs, undergoing *in vivo* reduction to their trivalent antimonial counterparts, which are responsible for the toxicity of antimonials against both the parasite and the human hosts.^35,36^ Therefore, trivalent antimony (Sb^III^) was used for the *in vitro* assessment of a possible synergism between **2** and antimonial compounds. The half-maximal inhibitory concentration against intracellular amastigotes of *L. infantum* (IC_50_) was used for such evaluation, employing five increasing concentrations of **2** (IC_50_ = 3.1 ± 1.3 μM), Sb^III^ (IC_50_ = 2.4 ± 0.47 μM), and the combination of both. Normalization of the data as a fractional response enabled performing calculations using a Bayesian model.^37^ The Bayesian inference allows asserting if a combination of compounds results in synergistic, antagonistic, or additive effects, by taking into account parameters such as the variability between different experiments, the variability within the same experiment (i.e., variability between replicates), and the variability among the observed responses of control experiments.^37^ Applying such a model to the data collected with **2**, Sb^III^, and their combination showed that both did not exert a synergistic effect *in vitro* against *L. infantum,* but rather an antagonistic one. Indeed, the combination resulted in a lower fractional response than both responses provided by **2** and Sb^III^ when assessed separately against *L. infantum* (Figure 3).

**Figure 3:**
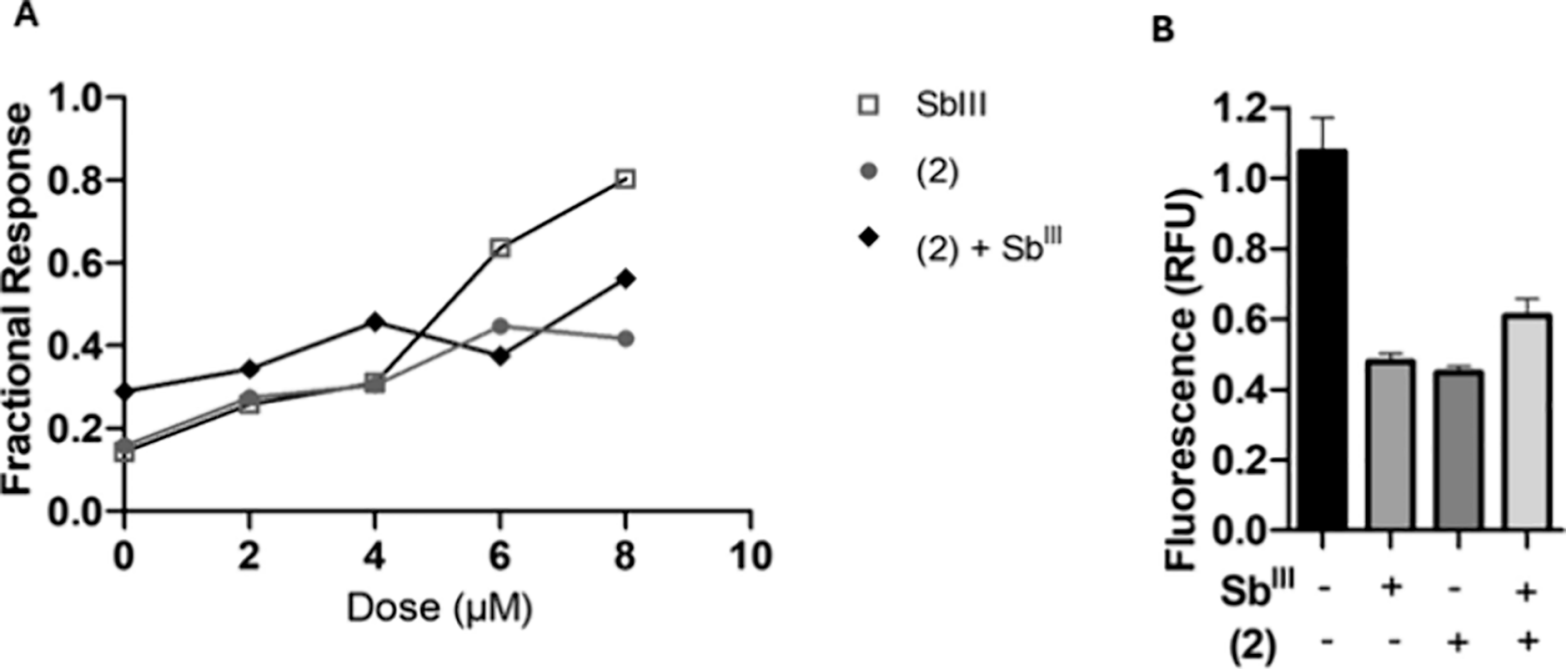
*In vitro* antileishmanial activity of **2**, Sb^III^ and their combination. The IC_50_ values were determined by nonlinear regression using a variable slope model with GraphPadPrism v8.2.0 (CA, USA) software. The data was normalized as a fractional response and a Bayesian model was applied to infer whether synergism, additivity or antagonism occurred between the two compounds. (A) Fractional response against *L. infantum::tdTomato*. (B) Fluorimetric quantification (RFU) of the inhibition of intracellular amastigotes. The results are representative of three independent experiments, each performed in duplicate.

#### Mode of Action

The potential mode of action of **2** was investigated by flow cytometry analysis, to examine the effects of this compound on the cellular functions of *L. infantum* promastigotes as well as its ability to generate reactive oxygen species in the intracellular environment. First, the antileishmanial activity of compound **2** against the promastigote form of *L. infantum* was assessed, revealing an IC_50_ of 7.93 μM against promastigotes, which corresponds to a potency approximately 2.5 times lower than against the amastigote form of the parasite (IC_50_ = 3.1 μM, Table 1).

Flow cytometry is a powerful technique for characterizing the effects of pharmaceutical compounds on the cellular functions of unicellular organisms or cells. One such technique is the cell cycle assay, which aims to identify specific phases of the cell cycle that are disrupted by drug candidates in trypanosomatids. This assay measures whether cells have halted their division process by evaluating DNA synthesis rates. Additionally, it enables the identification of cell death pathways, which may be either apoptotic or necrotic.^38,39^ To assess the effects of **2** and miltefosine (used as a reference drug) on the normal cell cycle progression of *L. infantum* promastigotes, the DNA content of treated and untreated cells was analyzed after 24 h of incubation. Cells were treated with compound concentrations of 4.0 μM (0.5 x IC_50_), 8.0 μM (IC_50_), and 16 μM (2 x IC_50_), and stained with Propidium Iodide (PI) before flow cytometry analysis.

The analysis of DNA content in treated and untreated cells (Figure 4) revealed a reduction in the number of cells, with no alteration in the cell cycle phases when compared to the untreated parasite control. A noticeable arrest in the G0/G1 phase was observed, where the majority of cells reside, indicating an increase in the size of cellular organelles, which characterizes this phase. Since these organelles duplicate once during the cell cycle, it was concluded that the extrusion of the daughter flagellum in promastigotes precedes mitosis, as seen in cytokinesis. Thus, such an arrest of the cell cycle during the G0/G1 phase may contribute to the observed decrease in parasite population, as reflected by the plots of growth kinetics. Therefore, it can be inferred that **2** may promote G0/G1 phase cell cycle arrest, resulting in an impaired growth and a lack of proliferation of the parasite. This also suggests that **2** may exhibit cytostatic rather than cytocidal effects.^40,41^

**Figure 4:**
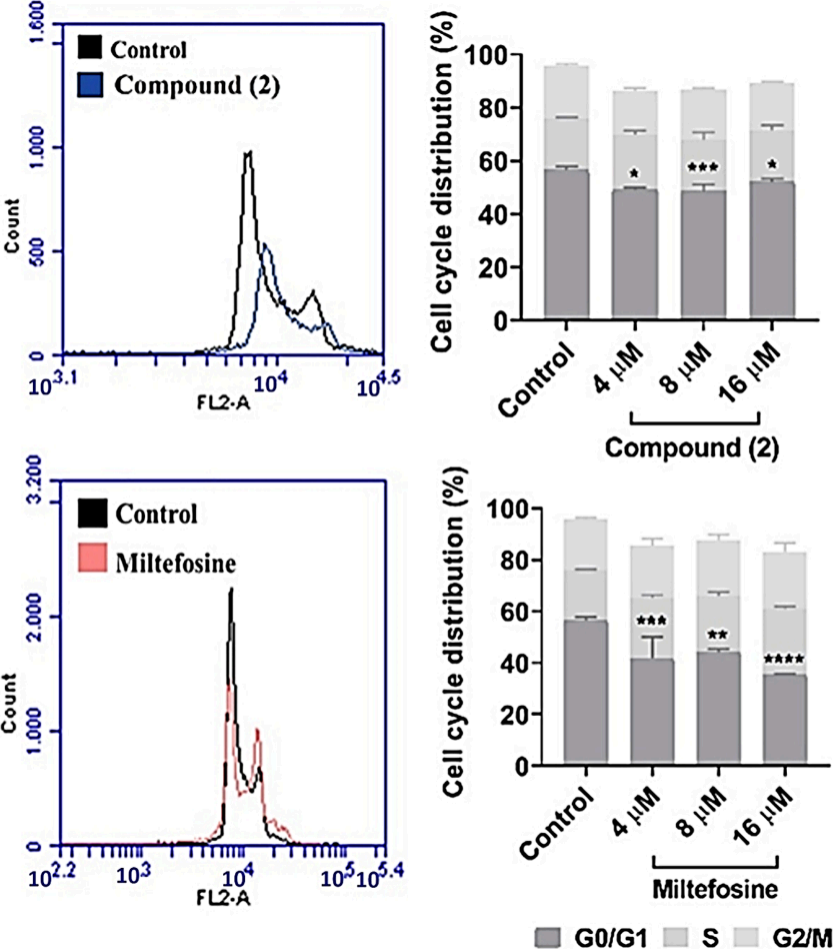
Cell cycle progression of *L. infantum* treated cells. Flow cytometry histograms of compound **2** and miltefosine. Parasites were cultured with compounds for 24 h. Untreated promastigote cells were used as negative control. Percentages correspond to the quantification of cells at each phase of the cell cycle. Cell counts in treated wells were compared to those in untreated wells using Dunnet test, and statistical significance was set at *p < 0.01, **p < 0.003, ***p < 0.0001 and ****p < 0.0001. Data shown is representative of three independent experiments.

This G0/G1 phase arrest, particularly in the G0 phase, is characteristic in apoptotic cells. Both **2** and miltefosine (used as a reference drug) showed this same pattern of cell cycle arrest, allowing both early and late apoptosis to be observed (Figure 5). Slater et al. suggested that the loss of cytoplasmic glutathione (GSH) is a key event in apoptosis, influencing the redox balance of cells and making them more susceptible to oxidative damage.^42^ A reduction in mitochondrial GSH levels can impair energy production, leading to cell death and progression to late apoptosis (Figure 5).

**Figure 5:**
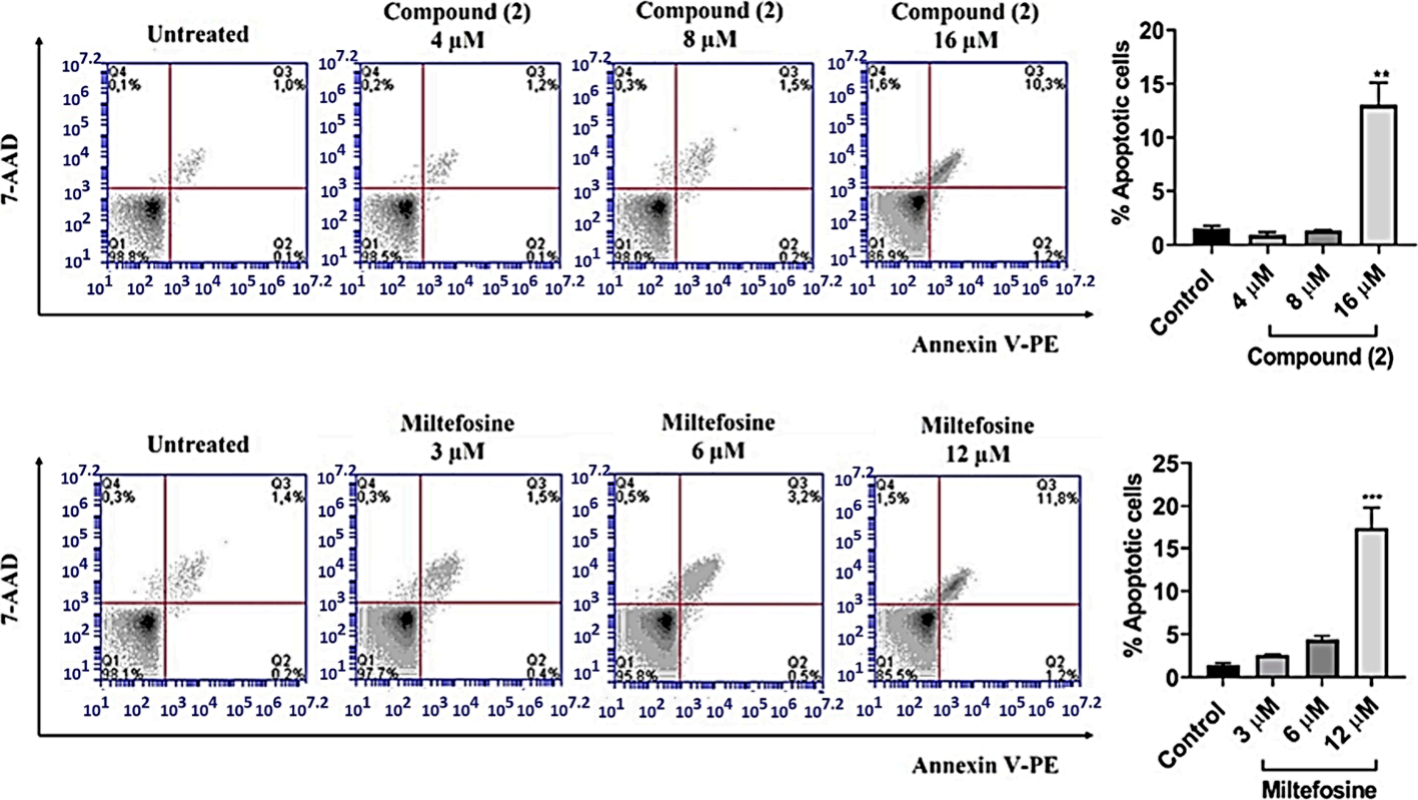
Flow cytometry histograms of **2** and miltefosine. The apoptotic rates were measured and analyzed by flow cytometry using Annexin V-FITC/PI staining in L. infantum treated cells. Parasites were cultured with compounds for 24 h. Untreated promastigotes cells were used as negative control. Percentages correspond to the quantification of cells at each apoptotic phase. Cell counts in treated wells were compared to those in untreated wells using Dunnett *t* test, and statistical significance was set at *p < 0.01, **p < 0.003, ***p < 0.0001 and ****p < 0.0001. Data shown is representative of three independent experiments.

This assertion is supported by the results from a reactive oxygen species (ROS) production assay. Mitochondrial dysfunction, caused by disturbances in the potential of the mitochondrial transmembrane and decreased ATP levels, promotes ROS generation, which plays a critical role in triggering late apoptosis.^43^ As shown in Figure 6, **2** induced a ROS production comparable to that of the standard H_2_O_2_, suggesting that ROS generation could be a key mechanism underlying the antileishmanial action of compound **2** and the observed inhibition of cell proliferation. Quite noteworthy, some fluorescence emission was detected in the negative control, which can be attributed to light scattering from the DMSO solvent used to dilute the samples.

**Figure 6:**
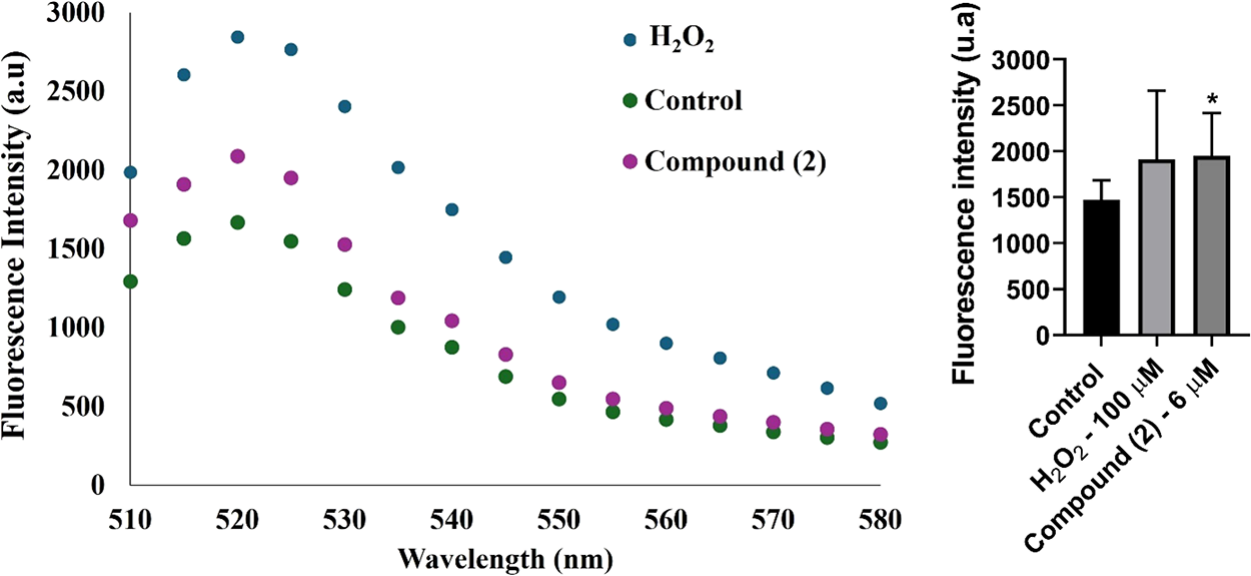
Effect of **2** on reactive oxygen species (ROS) generation in *L. infantum* in relation to the fluorescence generated and in relation to the percentage at each wavelength.

Taken together, these results suggest that **2** exerts a cytostatic effect on *L. infantum* promastigotes by promoting cell cycle arrest in the G0/G1 phase and triggering an increased ROS production.

#### Target Identification

##### In Silico Target-Fishing

Initial efforts toward the identification of the molecular target of **2** were carried out *in silico*, aiming to provide guidance for future lead optimization studies involving the pyrazinylpiperazine class, as well as for other antileishmanial target-based drug discovery efforts.

The molecular structure of **2** was examined with different bioinformatic approaches to screen both its metabolic pathway and its molecular target in *L. infantum* and *L. braziliensis*. SwissTarget, SEA, PharmMapper and SuperPred servers, which utilized chemical similarity to find proteins with known ligands, found 100, 48, 305, and 138 human molecular targets, respectively, while only 38 showed overlapping results for more than two servers (Figure S1A). Upon analyzing the homologous sequences in the proteomes of *L. infantum* and *L. braziliensis* using the BLASTp program, it was observed that 71 and 120 sequences, respectively, exhibited values beyond the cutoff. The resulting sequences were uploaded to the Cytoscape platform for functional enrichment analysis (Figure S1B), where the most enriched term for both parasite species was GO:0004674, which represents the *“protein serine/threonine kinase activity”* (Figure S1C,D). Similarly, both the second and third enriched terms GO:0016773 and GO:0016301 are associated with kinase activity. The kinomes of *L. infantum* and *L. braziliensis* were recently defined and compared using a bioinformatic pipeline,^44^ resulting in 29 kinase targets with orthology between the parasite species. The sequences of these targets were aligned for the visualization of conserved motifs (Figure S1E) and lately docked against **2** (Figure S1F). The results showed that **2** was predicted to bind to nine molecular kinase targets above the cutoff value, being a serine/threonine protein kinase (Uniprot ID: A4IAZ8) the one that showed the highest binding affinity (−6.68 kcal/mol).

Therefore, these *in silico* predictions suggest that the antileishmanial activity of **2** could be the result of the inhibition of a nonspecific serine/threonine protein kinase in *L. infantum*, disrupting the ATP binding/phosphoryltransferase activity in the parasite.^45^ Nonetheless, these *in silico* results need to be confirmed by *in vitro* experiments, given that the results provided by *in silico* target-fishing are of predictive rather than affirmative nature. Hence, inhibition assays with **2** against *Leishmania* kinases were planned, to provide conclusive elements regarding *in silico* predictions. Interestingly, a previous screening of potential glycogen synthase kinase-3 (GSK-3) inhibitors among the Leishbox showed that the initial hit **1** did not inhibit GSK-3, which is described as a multitask serine/threonine kinase in mammalian cells with close counterparts encountered in *Leishmania* species.^46^ Thus, the seemingly opposite conclusions between *in silico* predictions for **2** and previous research involving **1** motivated the *in vitro* assessment of the inhibitory activity of **2** against two GSK-3 isoforms encountered in *L. infantum* rather than other *Leishmania* kinases. Furthermore, given the relative lack of knowledge of the parasite’s biology and the overall lack of confirmed crystal structures for the vast majority of its proteins,^47^ potential targets may have been omitted by the *in silico* target-fishing, which led other *Leishmania* enzymes to be considered for the *in vitro* inhibition assays detailed in the next section. One of these, namely the heat shock protein 83 (Hsp83), despite not being a kinase itself, has been associated with protein kinase A activity in *Leishmania* parasites.^48^ Considering that *in silico* predictions associated protein kinase activity with a potential target, Hsp83 was therefore included in the panel of nonkinase *Leishmania* proteins to assess the inhibitory activity of **2**.

##### In Vitro Inhibition Assays

The *Leishmania* kinome consists of approximately 200 protein kinases, which play crucial roles in various cellular processes, including signaling, metabolism, and parasite survival. Among these, 43 have been identified as potentially essential for parasite proliferation, highlighting their significance as potential drug targets for therapeutic intervention.^44,49^ A previous genetic validation study has established that both glycogen synthase kinase-3 isoforms A (GSK-3A) and B (GSK-3B) are essential in *Leishmania* parasites.^49^ In order to assess the effect of **2** against *L. infantum* GSK-3A and *L. infantum* GSK-3B, the activity of recombinantly produced enzymes was monitored using a commercially available TR-FRET-based assay (LANCE TR-FRET, PerkinElmer). Compound **2** (0 – 30 μM) was titrated against fixed concentrations in protein (50 nM *L. infantum* GSK-3A or 10 nM *L. infantum* GSK-3B) and peptide substrate (50 nM). Data from enzyme activity assays showed that **2** had no inhibitory effect on *L. infantum* GSK-3A and GSK-3B enzymes at either concentration up to 30 μM. To provide further insight on the mechanisms of antileishmanial action of the pyrazinylpiperazine class, compounds **1** and **3** – **6** were also assessed against *L. infantum* GSK-3A and GSK-3B, following the same protocol as was used for the assessment of **2** against both isoforms. Such assessment revealed that, akin to **2**, the initial hit **1** and derivatives **3** – **6** did not exert any inhibition of *L. infantum* GSK-3A and GSK-3B at concentrations up to 30 μM.

Furthermore, **2** was subjected to biochemical and biophysical assays against a panel of *Leishmania* proteins to identify potential targets. Heat shock protein 83 (Hsp 83), superoxide dismutase (SOD), and dihydroorotate dehydrogenase (DHODH) from *L. braziliensis* were chosen for the assessment of the inhibitory activity of **2**. Hsp83 are evolutionarily conserved ATP-dependent proteins that play a crucial role in stabilizing and enhancing various client proteins, many of which are vital for ongoing cell signaling and adaptive stress responses.^50–52^ The expression of Hsps is crucial for the survival and protection of *Leishmania* in both life stages, as they confer thermotolerance, enhanced virulence, and improved adaptability to changes in pH, temperature, oxidative stress, and enzymatic manipulation.^53^ Consequently, inhibiting Hsp83 in *Leishmania* parasites can disrupt their survival in both stages of their life cycle and consequently hinder disease progression. Reactive oxygen species (ROS) play a role in intercellular signaling and in the synthesis of important biological molecules.^54,55^ Protozoan parasites such as *Leishmania* spp. have developed effective mechanisms to protect themselves against ROS, of which the first line of defense involves SOD (E.C. 1.15.1.1). This enzyme is responsible for converting superoxide into hydrogen peroxide and molecular oxygen.^56^ FeSOD activity is critical for the survival of *Leishmania* parasites within the host. The superoxide dismutase in *L. braziliensis* contains iron as a metal prosthetic group (FeSOD), while the human version uses copper and zinc (Cu/ZnSOD).^57,58^ This difference, along with their low sequence similarity, suggests that selective FeSOD inhibition in trypanosomatids is a viable therapeutic strategy.^59,60^ DHODH is a flavoenzyme that catalyzes the stereoselective oxidation of (*S*)-dihydroorotate (DHO) to orotate (ORO) in the fourth step of the *de novo* pyrimidine biosynthesis pathway. Previous studies have shown that knocking out the DHODH gene in *T. cruzi* results in cell nonviability,^61^ and silencing DHODH expression in *T. brucei* inhibits parasite growth.^62^

Generally, differential scanning fluorimetry (DSF) is used to identify the conditions and molecules that affect protein stability.^63^ This assay is based on the interaction of a fluorophore with the hydrophobic regions of a protein, which become exposed during thermal denaturation.^64^ The change in fluorescence corresponds to an increase in temperature and, consequently, protein unfolding. As a result, the mean thermal transition point (Tm, or melting temperature) can be calculated, allowing to observe how ligands may have an influence on the protein.^65^ The interaction between **2** and either *L. braziliensis* Hsp83 or *L. braziliensis* SOD was monitored by measuring the Tm value for each protein when exposed to compound **2** (100 μM) using DSF. Nevertheless, **2** failed to exert statistically significant effects, suggesting a weak interaction with these targets (Figure S24). In a similar manner to *L. infantum* GSK-3 inhibition, the inhibition of *L. braziliensis* Hsp83 and *L. braziliensis* SOD by the initial hit **1** and derivatives **3** – **6** was also assessed, to provide additional data regarding potential targets of the pyrazinylpiperazine class in *Leishmania* parasites. Titration curves for the binding of all compounds to each of these two enzymes were generated, using concentrations in compounds ranging from 100 μM to 12.5 μM. Dose–response data in DSF (Thermofluor) experiments is typically presented by showing the Tm shift as a function of ligand concentration.^66^ However, the data presented in Figure S25 does not exhibit such binding profile, suggesting that neither of these compounds interacts with either *L. braziliensis* Hso83 or *L. braziliensis* SOD.

Furthermore, DHODH inhibition assays resulted in less than 25% inhibition of this enzyme by **2** at a concentration as high as 100 μM, suggesting, in the same manner as previously observed with Hsp83 and SOD, a weak interaction with this target.

### *In Vivo* Antileishmanial Efficacy of 2 in a BALB/c Mice Model

#### Acute Toxicity and Maximum Tolerated Dose

Prior to the assessment of **2** in a visceral leishmaniasis mice model, its maximum tolerated dose (MTD) was determined in BALB/c mice, following recommended lead selection criteria for antileishmanial compounds.^67^ Two uninfected BALB/c mice (one male, one female) were administered intraperitoneally increasing doses of **2** (emulsion in polyethylene glycol) every 2 h, until a final accumulated dose of 100 mg/kg. The mice were inspected for clinical manifestations 24 h after the administration of the last dose of **2**, and the observation revealed no signs of acute toxicity in both animals. Therefore, the assessment of the *in vivo* antileishmanial activity of **2** in BALB/c mice was performed using doses of 50 mg/kg and 100 mg/kg.

#### Antileishmanial Activity in BALB/c Mice

We previously reported that transfection of *L. infantum* with the *tdTomato* gene does alter neither the infectivity of the parasite nor the efficacy of meglumine antimoniate for the treatment of animals infected with transfected *L. infantum::tdTomato* parasites.^68^ Therefore, the efficacy of **2** in a BALB/c mice model of visceral leishmaniasis was evaluated in mice infected with *tdTomato* mutant *L. infantum* lines, using meglumine antimoniate as a positive control.

Male BALB/c mice infected with *L. infantum* parasites were treated intraperitoneally with **2** (one group receiving a dose of 50 mg/kg/day and another a dose of 100 mg/kg/day), meglumine antimoniate (500 mg/kg/day) or left untreated (control group). Treatment began at 7 days postinfection (dpi) and lasted for 10 consecutive days. At 20 dpi (3 days after the end of the treatment), determination of the parasite burden in both the spleen and liver showed that treatment with **2** at 50 mg/kg/day reduced the parasitemia by 80.0% (*p* < 0.05) and 44.4% (not significant according to statistical analysis), respectively, when compared to the untreated control (Figure 7). Increasing the dose to 100 mg/kg/day of **2** satisfyingly promoted a significant improvement in parasite burden reduction, especially in the liver, reaching 91.1% (*p* < 0.01) and 90.0% (*p* < 0.01) parasitemia reduction in the spleen and liver, respectively. As expected, treatment with 500 mg/kg/day meglumine antimoniate almost completely eradicated *L. infantum* in both organs. No external signs of acute toxicity, such as ruffled fur, abnormal behavior or eye irritation, could be observed among both groups treated with **2** during and after the treatment period, suggesting that **2** does not exert acute toxicity *in vivo* when used at doses of 100 mg/kg/day or lower over moderate periods. No death was reported among the mice treated with **2** at either dose, making its toxicity profile extremely relevant for the treatment not only of VL but also of possibly other parasitic diseases, considering the relatively long treatment period (10 days) and daily dose (100 mg/kg). Therefore, treatment regimens with higher doses in **2** could be considered in future works, aiming to reduce treatment duration and/or completely reduce the parasite load in the liver and spleen of mice infected with *L. infantum*. From these results, confirming **2** as an effective antileishmanial lead, and considering that the superior permeability of **3** should improve its bioavailability and drug delivery while retaining anti-*L. infantum* activity equivalent to **2**, the methoxy derivative **3** emerges as a promising candidate for further *in vivo* evaluation, potentially acting as a prodrug of **2** from phase I metabolism.

**Figure 7:**
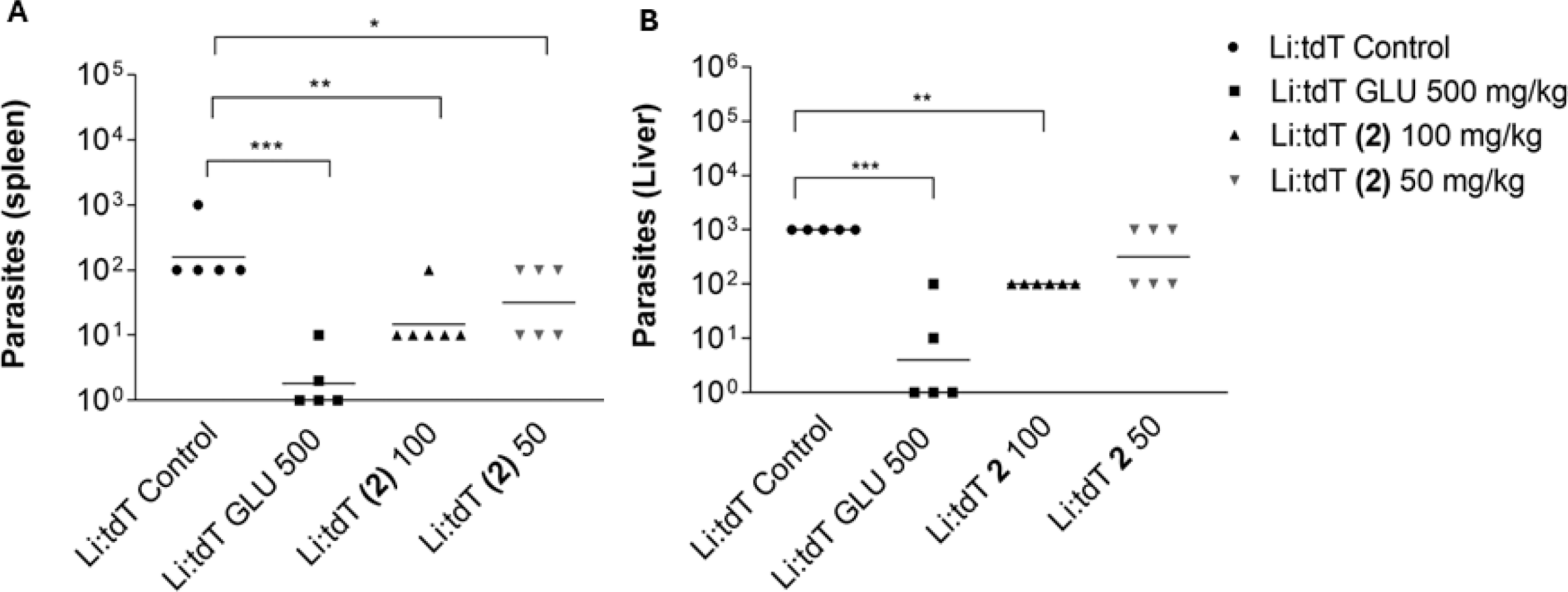
*In vivo* efficacy of **2** (50 mg/kg/day and 100 mg/kg/day) in a BALB/c mice VL model (10-day treatment). Meglumine antimoniate (500 mg/kg/day) was used as a positive control, and a group was left untreated as the negative control. The antileishmanial efficacy was evaluated through the number of viable parasites recovered from the spleen (A) and the liver (B) of mice treated with **2** and meglumine antimoniate, when compared to the untreated control (a logarithmic scale was used for the graphical representation). Horizontal bars represent the average number of viable parasites. Groups receiving treatment were compared to the negative control using one-way analysis of variance with Bonferroni *post hoc* test. Asterisks (*) represent significative differences relative to the untreated group (* *P* < 0.05; ** *P* < 0.01; *** *P* < 0.001; **** *P* < 0.0001). GLU: Meglumine antimoniate. *Li:tdT*: transfected *L. infantum::tdTomato* parasites.

## Conclusion

In conclusion, the *in vitro* assessment of the ADME profile of a select set of pyrazinylpiperazines previously identified as promising lead candidates allowed us to establish a small structure–property relationship which revealed that most compounds, except the catechol derivative **6**, displayed a satisfactory *in vitro* ADME profile considering their GI and BBB permeability, microsomal half-life, and intrinsic clearance. Based upon this structure–property relationship and its higher *in vitro* potency against *L. infantum* and low cytotoxicity toward host cells, the *para*-hydroxy derivative **2** was deemed the most suitable for *in vivo* assessment in a BALB/c mice model of visceral leishmaniasis, proving efficient in reducing the parasitemia by over 90% without inducing any acute toxicity when dosed at 100 mg/kg/day in a 7-day treatment. Ultimately, *in silico* target-fishing efforts toward the determination of the mechanism of action of **2** suggested a nonspecific serine/threonine protein kinase as a probable target in *L. infantum*, though *in vitro* inhibition assays carried out with *L. infantum* GSK-3A, GSK-3B, and a variety of other nonkinase *Leishmania* proteins could not manage to identify one of these as being the molecular target of **2**, highlighting the need for further enzymology-focused studies with this compound. Despite the current lack of clearly identified molecular target, flow cytometry and ROS production assays still managed to provide valuable mechanistic data, demonstrating that **2** promotes G0/G1 phase cell cycle arrest and increases ROS production in *L. infantum*. From this, it appears that the present work successfully identified **2** as a novel lead compound against visceral leishmaniasis, from which further lead optimization efforts involving pyrazinylpiperazines against visceral leishmaniasis should stem, focusing on increasing the GI permeability while retaining the adequate ADME profile and excellent *in vivo* antileishmanial activity.

## Materials and Methods

### Synthesis

#### General Information

Unless specified, all reactions were performed under magnetic stirring, and commercial reagents and solvents were used without further purification. Dichloromethane (DCM), triethylamine (Et_3_N) and dimethylformamide (DMF) were dried with molecular sieves. Ethyl acetate (EtOAc) and hexane (Hex) were previously distilled. Flash column chromatography was performed using either Aldrich silica gel (35–70 *mesh*) or Macherey-Nagel silica gel (230–400 mesh ASTM). Analytical thin-layer chromatography (TLC) was performed on chromatography aluminum sheets impregnated with silica-gel 60 F_254_ (Sigma-Aldrich) and plate revelation was achieved using UV light (254 nm) and/or iodine atmosphere. All reactions were monitored by TLC. ^1^H, proton-decoupled ^13^C and ^13^C APT NMR spectra were acquired in CDCl_3_ or DMSO-*d*_*6*_ at 400 MHz (^1^H) and 101 MHz (^13^C and ^13^C APT) (Bruker Ascend 400). Chemical shifts (δ) are reported in ppm using residual solvent peak as an internal standard (CDCl_3_: 7.26 ppm, DMSO-*d*_*6*_: 2.50 ppm, TMS: 0.00 ppm for ^1^H NMR spectra, and CDCl_3_: 77.16 ppm, DMSO-*d*_*6*_: 39.52 ppm for ^13^C and ^13^C APT NMR spectra). Peak multiplicity was reported using the following abbreviations: s = singlet, d = doublet, t = triplet, q = quartet, dd = doublet of doublets, m = multiplet. The multiplicity is followed by the coupling constant(s) in Hz and integration. Exchangeable protons were not observable on some NMR spectra. For ^13^C APT NMR spectra, peaks pointing upward correspond to primary and tertiary carbons, and peaks pointing downward correspond to secondary and quaternary carbons. High resolution mass spectrometry (HRMS) was measured using electrospray ionization (ESI) (Bruker Daltonics Corporation, Q-TOF geometry Impact II). The NMR and HRMS spectra of compounds **1** – **6** are available in the Supporting Information. The purity of all significant compounds in this work was confirmed to be >95% by HPLC analysis.

#### Synthesis of the 2-(Piperazin-1-yl)-pyrazine Intermediate **8**

2-chloropyrazine (300 mg, 2.619 mmol) was solubilized in iPrOH (0.5 M). Piperazine (676.8 mg, 7.857 mmol, 3 equiv) and Na_2_CO_3_ (832.7 mg, 7.857 mmol, 3 equiv) were added, and the reaction was stirred for 15 h at 80 °C. iPrOH was evaporated under reduced pressure, and the crude residue was purified by flash column chromatography (DCM/MeOH (9:1) to (8:2) gradient).

**8**: yellow oil, 435.7 mg, 100%. ^**1**^ **H NMR (400 MHz, CDCl**_**3**_ **):** δ 8.11 (d, *J* = 1.2 Hz, 1H), 8.04 (dd, *J* = 2.7, 1.4 Hz, 1H), 7.82 (d, *J* = 1.6 Hz, 1H), 3.59 – 3.49 (m, 4H), 3.03 – 2.91 (m, 4H), 1.93 (s, 1H). ^**13**^ **C NMR (101 MHz, CDCl**_**3**_ **):** δ 155.32, 141.84, 132.99, 131.14, 45.84, 45.65.

#### Synthesis of Amides **1** – **5** and **7**

All the amides **1 – 5** and **7** were synthesized according to the following General Procedure:

The appropriate carboxylic acid (1.2 equiv), EDC (1.3 equiv) and HOBt (0.1 equiv) were solubilized in DMF (0.1 – 0.3 M), **8** (1 equiv) was added and the solution was stirred at room temperature until completion of the reaction. The reaction was quenched with cold water, and the aqueous layer was extracted with EtOAc (3 × 15 mL) (for compound **5**, the precipitate was filtered, washed with cold water and dried *in vacuo*). The combined organic layers were washed with brine, dried over Na_2_SO_4_, filtered, and the solvent was evaporated under reduced pressure. The crude residue was then purified by flash column chromatography to yield the target compound.

#### (3-Chloro-4-hydroxyphenyl)­(4-(pyrazin-2-yl)­piperazin-1-yl)­methanone (1)

Following the General Procedure, from **8** (20 mg, 0.122 mmol) and 3-chloro-4-hydroxybenzoic acid (25.3 mg, 0.146 mmol), using a mix of EtOAc/Hex (95:5) for the flash column chromatography.

**1**: white solid, 19.5 mg, 50%. ^**1**^ **H NMR (400 MHz, CDCl**_**3**_ **)** δ 8.10 (s, 1H), 8.07 (m, 1H), 7.85 (d, *J* = 2.5 Hz, 1H), 7.41 (d, *J* = 1.8 Hz, 1H), 7.20 (dd, *J* = 8.3, 1.8 Hz, 1H), 6.94 (d, *J* = 8.3 Hz, 1H), 3.75 – 3.60 (m, 8H). ^**13**^ **C NMR (101 MHz, CDCl**_**3**_ **)** δ 169.23, 154.89, 153.18, 141.97, 134.04, 131.31, 128.92, 128.64, 128.04, 120.38, 116.40, 44.83. **HRMS (ESI + ):** calcd for C_15_H_15_ClN_4_O_2_Na^+^ [M + Na]^+^ = 341.0776 *m*/*z*, found [M + Na]^+^ = 341.0773 *m*/*z*, error = 0.88 ppm.

#### (4-Hydroxyphenyl)­(4-(pyrazin-2-yl)­piperazin-1-yl)­methanone (2)

Following the General Procedure, from **8** (20 mg, 0.122 mmol) and 4-hydroxybenzoic acid (20.2 mg, 0.146 mmol), using a mix of EtOAc/Hex (95:5) for the flash column chromatography.

**2**: white/yellowish solid, 19.5 mg, 56%. ^**1**^ **H NMR (400 MHz, CDCl**_**3**_ **)** δ 8.15 (s, 1H), 8.10 (dd, *J* = 2.6, 1.5 Hz, 1H), 7.91 (d, *J* = 2.6 Hz, 1H), 7.77 (br. s, 1H), 7.31 (d, *J* = 8.6 Hz, 2H), 6.81 (d, *J* = 8.6 Hz, 2H), 3.71 (m, 8H). ^**13**^ **C NMR (101 MHz, CDCl**_**3**_ **)** δ 171.38, 158.61, 154.95, 142.11, 133.67, 131.07, 129.51, 126.48, 115.73, 44.81. **HRMS (ESI + ):** calcd for C_15_H_16_N_4_O_2_Na^+^ [M + Na]^+^ = 307.1165 *m*/*z*, found [M + Na]^+^ = 307.1163 *m*/*z*, error = 0.65 ppm. **Purity:** 97.3% (HPLC).

#### (4-Methoxyphenyl)­(4-(pyrazin-2-yl)­piperazin-1-yl)­methanone (3)

Following the General Procedure, from **8** (20 mg, 0.122 mmol) and 4-methoxybenzoic acid (22.2 mg, 0.146 mmol), using a mix of EtOAc/Hex (9:1) for the flash column chromatography.

**3**: yellow oil, 16.3 mg, 45%. ^**1**^ **H NMR (400 MHz, CDCl**_**3**_ **)** δ 8.16 (s, 1H), 8.08 (s, 1H), 7.90 (s, 1H), 7.42 (dd, *J* = 8.8, 4.6 Hz, 2H), 6.93 (dd, *J* = 8.8, 4.6 Hz, 2H), 3.84 (s, 3H), 3.70 (m, 8H). ^**13**^ **C NMR (101 MHz, CDCl**_**3**_ **)** δ 170.79, 161.22, 154.97, 141.98, 133.78, 131.22, 129.40, 127.50, 114.02, 55.53, 44.83. **HRMS (ESI + ):** calcd for C_16_H_19_N_4_O_2_^+^ [M + H]^+^ = 299.1502 *m*/*z*, found [M + H]^+^ = 299.1502 *m*/*z*, error = 0.00 ppm.

#### (6-Aminopyridin-3-yl)­(4-(pyrazin-2-yl)­piperazin-1-yl)­methanone (4)

Following the General Procedure, from **8** (20 mg, 0.122 mmol) and 6-aminonicotinic acid (20.2 mg, 0.146 mmol), using a mix of DCM/MeOH (9:1) for the flash column chromatography.

**4**: yellow solid, 26.7 mg, 77%; ^**1**^ **H NMR (400 MHz, CDCl**_**3**_ **)** δ 8.23 (dd, *J* = 2.3, 0.7 Hz, 1H), 8.16 (d, *J* = 1.5 Hz, 1H), 8.09 (dd, *J* = 2.6, 1.5 Hz, 1H), 7.91 (d, *J* = 2.6 Hz, 1H), 7.60 (dd, *J* = 8.5, 2.3 Hz, 1H), 6.52 (dd, *J* = 8.5, 0.8 Hz, 1H), 4.75 (s, 2H), 3.86 – 3.51 (m, 8H). ^**13**^ **C NMR (101 MHz, CDCl**_**3**_ **)** δ 169.14, 159.56, 154.88, 148.22, 141.93, 138.11, 133.96, 131.28, 121.06, 108.05, 44.82. **HRMS (ESI + ):** calcd for C_14_H_17_N_6_O^+^ [M + H]^+^ = 285.1458 *m*/*z*, found [M + H]^+^ = 285.1453 *m*/*z*, error = 1.75 ppm. **Purity:** 100.0% (HPLC).

#### (6-Hydroxynaphthalen-2-yl)­(4-(pyrazin-2-yl)­piperazin-1-yl)­methanone (5)

Following the General Procedure, from **8** (20 mg, 0.122 mmol) and 6-hydroxy-2-naphthoic acid (27.5 mg, 0.146 mmol). Filtration of the yellowish precipitate, washing with cold water and drying *in vacuo* afforded **5** without any further purification needed.

**5**: white/yellowish solid, 37.8 mg, 93%. ^**1**^ **H NMR (400 MHz, DMSO-** *d*_**6**_ **)** δ 9.94 (s, 1H), 8.34 (d, *J* = 1.3 Hz, 1H), 8.10 (dd, *J* = 2.6, 1.5 Hz, 1H), 7.90 (d, *J* = 0.8 Hz, 1H), 7.86 (m, 2H), 7.75 (d, *J* = 8.5 Hz, 1H), 7.44 (dd, *J* = 8.5, 1.7 Hz, 1H), 7.15 (m, 2H), 3.66 (s, 8H). ^**13**^ **C NMR (101 MHz, DMSO-** *d*_**6**_ **)** δ 169.54, 156.43, 154.44, 141.42, 135.01, 132.74, 131.44, 130.06, 129.62, 126.80, 126.69, 126.09, 124.83, 119.38, 108.62, 43.95. **HRMS (ESI + ):** calcd for C_19_H_18_N_4_O_2_Na^+^ [M + Na]^+^ = 357.1322 *m*/*z*, found [M + Na]^+^ = 357.1319 *m*/*z*, error = 0.84 ppm. **Purity:** 95.7% (HPLC).

#### (2,3-Dimethoxyphenyl)­(4-(pyrazin-2-yl)­piperazin-1-yl)­methanone (7)

Following the General Procedure, from **8** (20 mg, 0.122 mmol) and 2,3-dimethoxybenzoic acid (26.6 mg, 0.146 mmol), using a mix of EtOAc/Hex (95:5) for the flash column chromatography.

**7**: yellowish oil, 32.4 mg, 83%. ^**1**^ **H NMR (400 MHz, CDCl**_**3**_ **)** δ 8.13 (d, *J* = 1.2 Hz, 1H), 8.09 – 8.03 (m, 1H), 7.88 (d, *J* = 2.6 Hz, 1H), 7.12 (t, *J* = 7.9 Hz, 1H), 6.96 (dd, *J* = 8.2, 1.2 Hz, 1H), 6.85 (dd, *J* = 7.6, 1.3 Hz, 1H), 4.07 (m, 1H), 3.90 (s, 3H), 3.86 (s, 2H), 3.80 (m, 2H), 3.69 – 3.59 (m, 1H), 3.56 (m, 2H), 3.46 (m, 1H), 3.37 – 3.26 (m, 1H). ^**13**^ **C NMR (101 MHz, CDCl**_**3**_ **)** δ 167.82, 154.91, 152.80, 145.13, 141.97, 133.68, 131.21, 130.96, 125.12, 119.36, 113.33, 61.78, 55.98, 46.57, 45.15, 44.56, 41.48. **HRMS (ESI + ):** calcd for C_17_H_20_N_4_O_3_Na^+^ [M + Na]^+^ = 351.1427 *m*/*z*, found [M + Na]^+^ = 351.1427 *m*/*z*, error = 0.00 ppm.

### Synthesis of (2,3-Dihydroxyphenyl)­(4-(pyrazin-2-yl)­piperazin-1-yl)­methanone (**6**)

**7** (15.6 mg, 0.048 mmol) was solubilized in DCM (0.5 M) under Ar atmosphere, and the solution was cooled down to 0 °C. BBr_3_ (0.480 mmol, 10 eq, 1 M solution in DCM) was added dropwise under stirring and Ar atmosphere, and the solution was stirred at room temperature. Upon completion of the reaction, cold water was added dropwise at 0 °C, and the aqueous layer was extracted with DCM (3 × 15 mL). The combined organic layers were washed with brine, dried over Na_2_SO_4_, filtered, and the solvent was evaporated under reduced pressure. The crude residue was then purified by flash column chromatography (DCM/MeOH (92:8)) to yield **6**.

**6**: yellowish solid, 6.0 mg, 42%. ^**1**^ **H NMR (400 MHz, CDCl**_**3**_ **)** δ 8.12 (s, 1H), 8.08 (s, 1H), 7.86 (s, 1H), 6.94 (dd, *J* = 5.9, 3.4 Hz, 1H), 6.83 – 6.72 (m, 2H), 3.86 – 3.76 (m, 4H), 3.73 – 3.60 (m, 4H). ^**13**^ **C NMR (101 MHz, CDCl**_**3**_ **)** δ 170.42, 154.84, 145.86, 144.77, 142.13, 133.36, 130.87, 119.84, 119.14, 119.05, 117.62, 44.63. **HRMS (ESI + ):** calcd for C_15_H_16_N_4_O_3_Na^+^ [M + Na]^+^ = 323.1115 *m*/*z*, found [M + Na]^+^ = 323.1112 *m*/*z*, error = 0.93 ppm. **Purity:** 100.0% (HPLC).

### Optimization of the Synthesis of **2** on a Larger Scale

#### 4-Acetoxybenzoic Acid (9)

4-hydroxybenzoic acid (1 g, 7.24 mmol) was solubilized in acetic anhydride (3.43 mL, 3.70 g, 36.28 mmol, 5 equiv), H_2_SO_4_ (96%) (0.1 mL, cat.) was added, and the reaction was stirred for 16 h at 80 °C. The reaction mixture was then cooled down to 0 °C, slowly diluted with water (30 mL), and the resulting precipitate was filtered, thoroughly washed with cold water and dried *in vacuo* to afford **9** as a brown solid.

**9**: brown solid, 1.31 g, 100%. ^**1**^ **H NMR (400 MHz, CDCl**_**3**_ **)** δ 8.14 (d, *J* = 8.8 Hz, 2H), 7.21 (d, *J* = 8.8 Hz, 2H), 2.33 (s, 3H). ^**13**^ **C NMR (101 MHz, CDCl**_**3**_ **)** δ 170.63, 168.96, 155.11, 131.98, 127.03, 121.89, 21.28.

#### 4-(4-(Pyrazin-2-yl)­piperazine-1-carbonyl)­phenyl Acetate (10)

**9** (374.7 mg, 2.08 mmol, 2 equiv) was solubilized in anhydrous DCM (10.4 mL) under Ar atmosphere, oxalyl chloride (1.32 g, 10.4 eq, 10 equiv) and DMF (3 drops, cat.) were carefully added at 0 °C, and the reaction was stirred for 2 h at 0 °C under Ar atmosphere. The volatiles were evaporated under reduced pressure, and the residue was solubilized in anhydrous DCM (5.2 mL) and carefully added to a stirred mixture of **8** (170 mg, 1.04 mmol) and Et_3_N (315.7 mg, 3.12 mmol, 3 equiv) at 0 °C, under Ar atmosphere. The reaction was then allowed to gradually warm up to room temperature and stirred for 3 h at room temperature under Ar atmosphere. The reaction mixture was then diluted with DCM (20 mL) and washed with water and brine (3 × 10 mL), and the aqueous layer was back-extracted with DCM (3 × 10 mL). The combined organic layers were dried over anhydrous Na_2_SO_4_, filtered, and the solvent was evaporated under reduced pressure. The residue was then purified by flash column chromatography (EtOAc/Hex (95:5)) to yield **10**.

**10**: white/yellowish solid, 226.6 mg, 67%. ^**1**^ **H NMR (400 MHz, CDCl**_**3**_ **)** δ 8.17 (d, *J* = 1.2 Hz, 1H), 8.09 (dd, *J* = 2.5, 1.2 Hz, 1H), 7.92 (d, *J* = 2.5 Hz, 1H), 7.48 (d, *J* = 8.5 Hz, 2H), 7.17 (d, *J* = 8.5 Hz, 2H), 3.93 – 3.58 (m, 8H), 2.32 (s, 3H). ^**13**^ **C NMR (101 MHz, CDCl**_**3**_ **)** δ 169.95, 169.20, 154.89, 152.05, 141.95, 133.97, 132.96, 131.28, 128.81, 122.02, 44.84, 21.27.

#### (4-Hydroxyphenyl)­(4-(pyrazin-2-yl)­piperazin-1-yl)­methanone (2)

**10** (215 mg, 0.659 mmol) was solubilized in MeOH/THF (3:1) (2 mL), NaOH (2 M) (4.95 mL, 9.89 mmol, 15 equiv) was added, and the reaction was stirred for 3 h at room temperature. The volatiles were distilled off under reduced pressure, and HCl (2 M) was added dropwise at 0 °C until pH ∼ 2. The pH was then adjusted to ∼ 8 with NaOH (2 M), and the aqueous layer was extracted with EtOAc (3 × 15 mL). The combined organic layers were dried over anhydrous Na_2_SO_4_, filtered, and the solvent was evaporated under reduced pressure to yield **2**.

**2**: white/yellowish solid, 185.7 mg, 99% (66% over three steps from 4-hydroxybenzoic acid). Analytical data: see above.

### High-Resolution Mass Spectrometry (HRMS)

High-resolution mass spectrometry (HRMS) was performed on a Q-TOF geometry Impact II spectrometer (Bruker Daltonics Corporation, Germany) equipped with an electrospray ionization source (ESI) and operated in positive ion mode. Parameters were set as follows for the analysis of each sample: capillary voltage: 4500 V, with an end plate offset potential of 500 V; nebulizer pressure: 0.4 bar; dry gas flow: 4 L/min; dry temperature: 180 °C. Spectra were acquired with an acquisition rate of 1.00 Hz, monitoring a mass range from 50 to 700 *m*/*z*. A syringe pump (KDS Legato 100, KD Scientific, Holliston, MA, USA) was used at a flow rate of 10 μL/min to infuse the calibrant solution of sodium formate (10 mmol L^–1^; isopropanol:water; 1:1; v:v).

### Purity Analysis by High Performance Liquid Chromatography (HPLC)

High performance liquid chromatography (HPLC) was performed on a Shimadzu Prominence liquid chromatography device (Shimadzu Corporation, Japan) equipped with an UV–vis detector, coupled to an ion trap mass spectrometer (Bruker Amazon SL, Bruker Amazon SL, Bruker Daltonics Corporation, Germany). The compound sample was solubilized in LC-MS grade MeOH (500 μL), and 10 μL of the resulting solution were injected into the chromatography device. The analysis was run on an Eclipse plus C18 column (250 × 4.6 mm, 5 μm) using water (A) and MeOH + 0.1% formic acid (B) as the mobile phase, eluting with an isocratic elution of A/B (30:70) (v/v). The analysis was performed at a stabilized temperature (35 °C) and a flow rate of 0.7 mL/min, and detection was achieved at a wavelength of 254 nm. After UV–vis detection, the analytes were analyzed by mass spectrometry, operating in positive mode. Data analysis was performed using the Bruker DataAnalysis 5.2 software (Bruker Daltonics Corporation, Germany).

### *In Vitro* Biological Assays

The *in vitro* anti-*L. infantum* activity (IC_50_) of each compound was evaluated on THP-1 cells (human leukemia monocytic cell line) infected with *L. infantum* promastigotes, and the *in vitro* cytotoxicity (CC_50_) was determined over the THP-1 cell line. The selectivity index (SI) was calculated as the ratio of the CC_50_ value determined in the cytotoxicity assay, divided by the IC_50_ value determined in the antileishmanial activity assay.

#### Parasitology Assays

##### Antileishmanial Activity Assay

Cells derived from the human monocytic strain THP-1 were cultured in complete Rowell Park Memorial Institute (RPMI)-1640 medium (supplemented with 10% fetal bovine serum, 100 U/mL penicillin, and 100 μg/mL streptomycin). Monocytes were differentiated into macrophages by the addition of 20 ng/mL phorbol myristate acetate (PMA). After 72 h, the macrophages (5 × 10^4^), in black 96-well microtiter plates (Corning Incorporated, Corning, NY), were infected with *Leishmania (Leishmania) infantum* (MHOM/BR/1974/PP75) promastigotes transfected with gene *tdTomato*, on the second day of the stationary phase (20 parasites per macrophage) for 4 h. The parasites that failed to infect the macrophages were washed away (three times with 1X PBS), and the infected macrophages were incubated for 72 h in RPMI-1640 medium containing different compound concentrations (80, 40, 20, 10, and 5 μg/mL). The compound concentration that inhibits 50% of parasite growth (IC_50_) was determined by the decrease in parasite fluorescence in the absence and presence of the compound, after 72 h of exposure, using a microplate reader (Varioskan LUX, Thermoscientific) with excitation and emission values: 554 and 581 nm, respectively. The IC_50_ values were determined by nonlinear regression using a variable slope model (log­(concentration) vs growth inhibition) with the GraphPadPrism v8.2.0 (CA, USA) software. Controls with uninfected cells, untreated infected cells, and infected cells treated with amphotericin B at 0.25 μM (positive control) or DMSO 1% were used. Quadruplicates were run on the same plate, and the experiments were repeated at least once.

##### Cytotoxicity Assay in Macrophages Derived from THP-1 Cells

The human monocytic THP-1 cells were differentiated into macrophages in 96-well microtiter plates for 72 h, according to the description above. The medium was then replaced, and the cells were exposed to the compounds at increasing concentrations starting at IC_50_ value against *L. infantum*. After 72 h of incubation with the compounds, AlamarBlue was added and the absorbance at 570 and 600 nm was measured after 4h. Controls with untreated and DMSO 1%-treated cells were run in parallel. The results were expressed as the percentual difference in the reduction between treated and untreated cells. The compound concentration that inhibits 50% of the THP-1 cell viability (CC_50_) was determined. The CC_50_ values were determined by nonlinear regression using a variable slope model (log­(concentration) vs growth inhibition) with the GraphPadPrism v8.2.0 (CA, USA) software. Triplicates were run on the same plate and the experiments were repeated at least once.

##### Synergism between **2** and Antimonials

Cells derived from the human monocytic strain THP-1 were cultured in complete Rowell Park Memorial Institute (RPMI)-1640 medium (supplemented with 10% fetal bovine serum, 100 U/mL penicillin, and 100 μg/mL streptomycin). Monocytes were differentiated into macrophages by the addition of 20 ng/mL phorbol myristate acetate (PMA). After 72 h, the macrophages (5 × 10^4^), in black 96-well microtiter plates (Corning Incorporated, Corning, NY), were infected with *Leishmania (Leishmania) infantum* (MHOM/BR/1974/PP75) promastigotes expressing red fluorescence protein on the second day of the stationary phase (20 parasites per macrophage) for 4 h. The parasites that failed to infect the macrophages were washed away (three times with 1X PBS). To investigate the possibility of synergism between Sb^III^ and compound **2**, 6 different concentrations of these drugs were used (1.0, 0.5, 0.2, 0.1, and 0.05 μg/mL), the solutions of Sb^III^ and **2** being prepared and diluted the day of the assay. The compounds were tested separately and in combination. The effective compound concentration that inhibits 50% of parasite growth (EC_50_) was determined by the decrease in parasite fluorescence after 72 h of exposure, in the absence and presence of the compound, using a microplate reader (Varioskan LUX, Thermoscientific) with excitation and emission values set at 554 and 581 nm, respectively. The EC_50_ values were determined by nonlinear regression using a Variable slope model (log­(concentration) vs growth inhibition) with the GraphPadPrism v8.2.0 (CA, USA) software. By normalizing the data as a fractional response, it was possible to perform the calculation using a Bayesian model.^37^ The results were compared to each other to determine whether the combination of compounds has a synergistic, antagonistic or additive effect. Controls with uninfected cells, untreated infected cells, infected cells treated with amphotericin B at 0.25 μM (positive control) or DMSO 1% were used. Quadruplicates were run on the same plate, and the experiments were repeated at least once.

##### Antileishmanial Activity Assay against Promastigotes

*L. infantum* promastigotes (MHOM/MA/67/ITMAP-263) were cultured in complete Schneider medium (SigmaAldrich, St. Louis, MO, USA), which consisted of Schneider medium plus 10% heat-inactivated fetal bovine serum (FBS, SigmaAldrich) pH 7.4, at 24 °C. The *L. infantum* promastigotes were plated at a concentration of 10^5^ parasites/mL, and **2** was added at concentrations of 64, 32, 16, 8, 4, 2, 1, 0.5, 0.25, and 0.125 μM. The cells were cultured for 72 h and, after the incubation time, cell proliferation was measured by colorimetric MTS assay. The cells in each well were stained with 20 μL of MTS (Promega, G3580) for 3 h, and the OD at 490 nm was determined with a microplate reader. All experiments were independently repeated at least three times.^69^

#### ADMET Experiments

##### Parallel Artificial Membrane Permeability Assay (PAMPA) – Blood-Brain Barrier (BBB)

One mg of each compound was solubilized in ethanol (1 mL) in a 5 mL flask, and an additional 500 μL ethanol and 3.5 mL PBS buffer (pH 7.4) were added to this solution. The resulting solution was then filtered through a 0.45 μM polyvinylidene fluoride (PVDF) filter, and the filtrate was kept. 180 μL of a PBS (pH 7.4)/ethanol (70:30) solution were then added to the wells of a 96-well receiver plate, and 5 μL of a porcine brain lipid solution (20 mg/mL in dodecane) to the wells of a 96-well donor plate. After 5 min, 180 μL of the solutions containing each compound were added to the wells of the donor plate, in triplicate, and the donor plate was carefully placed over the receiver plate in a “sandwich-like” system, which was left standing for 2 h 45 min at rt in a sealed vessel containing 10 mL PBS (pH 7.4). Afterward, the donor plate was removed, and the content of the receiver plate was transferred to a UV reading plate, which was read using a microplate reader (SpectraMax 5 – Molecular Devices). A reference sample for the internal correction of the microplate reader was prepared using 180 μL of the PBS (pH 7.4)/ethanol (70:30) solution (adapted from ref 23). The optical density values obtained from the content of the receiver plate were compared with the ones obtained from readings performed with the initial solutions containing each compound, before their addition to the donor plate. Triplicates were run in the same plate, and the experiments were run at least twice, using the following experimental controls: atenolol, caffeine, diazepam, enoxacin, ofloxacin, testosterone and verapamil, which are reference compounds with different reported permeability values used as an experimental standard.^24^ The optical density values for the experimental controls were used to determine the equation of the linear regression (optical density *vs P*_*e*_), which allowed the determination of the *P*_*e*_ value of each compound from the optical density values obtained from the receiver plate, using an in-house Microsoft Excel spreadsheet.

##### Parallel Artificial Membrane Permeability Assay (PAMPA) – Gastrointestinal Tract (GI)

A 10 mM solution of each compound in DMSO was prepared and subsequently homogenized in a 5 mL flask with 4.75 mL PBS (pH 6.6) (10 mM). The resulting solution was then filtered through a 0.45 μM PVDF filter, and the filtrate was kept. 180 μL of a PBS (pH 7.4)/DMSO (95:5) solution were then added to the wells of a 96-well receiver plate, and 5 μL of a L-α-phosphatidylcholine solution (20 mg/mL in dodecane) to the wells of a 96-well donor plate. After 5 min, 180 μL of the solutions containing each compound were added to the wells of the donor plate, in triplicate, and the donor plate was carefully placed over the receiver plate in a “sandwich-like” system, which was left stirring (50 rpm) for 8 h at rt in a sealed vessel containing 10 mL PBS (pH 7.4). Afterward, the donor plate was removed, and the content of the receiver plate was transferred to a UV reading plate, which was read using a microplate reader (SpectraMax 5 – Molecular Devices). A reference sample for the internal correction of the microplate reader was prepared using 180 μL of the PBS (pH 7.4)/DMSO (95:5) solution (adapted from refs 70 and 71). The optical density values obtained from the content of the receiver plate were compared with the ones obtained from readings performed with the initial solutions containing each compound, before their addition to the donor plate. Triplicates were run in the same plate, and the experiments were run at least twice, using the following experimental controls: acyclovir, atenolol, ceftriaxone, coumarin, diclofenac, hydrocortisone, norfloxacin, ranitidine, sulfasalazine and verapamil, which are reference compounds with different reported permeability values used as an experimental standard.^72^ The optical density values from the receptor plate were compared to those from experimental controls to determine the *F*_*a*_ value for each compound, using Microsoft Excel.

##### Metabolic Stability in Mouse Liver Microsomes (MLM)

To a microcentrifuge tube was added 1 mg/mL Mouse Liver Microsome (MLM), a NADPH generating system consisting of 1 mM NADP+, 1.5 mM MgCl_2_, 3.5 mM glucose-6-phosphate and 0.5 U/mL glucose-6-phosphate dehydrogenase, and phosphate buffer (pH 7.4) in a sufficient amount for a total volume of 248.75 μL. After a 15 min preincubation at 37 °C in a water bath, the enzymatic reactions were started by adding 2 μL of a 500 μM stock solution of each compound to be assessed, reaching a final concentration of 5 μM in the tested compound.^73,74^ The samples were further incubated at 37 °C under stirring (30 rpm) for predetermined periods (0, 15, 30, 45, and 60 min), and 500 μL chilled MeOH and 500 μL chilled acetonitrile were added before the samples were homogenized and left standing in an ice bath to accelerate protein precipitation. The resulting mixture was centrifugated at 13000 rpm for 15 min at 4 °C, the organic layer was extracted and 2 μL internal standard (clonazepam 5 μM) was added. Finally, the samples were filtered through a PVDF filter (Millex 0.22 μm x 13 mm) and analyzed by HPLC-PDA.^23,73,74^

Four experimental controls were used for all analyses: a reference sample of the enzymatic medium without the tested compound, to identify the HPLC peaks corresponding to the enzymatic medium; a sample containing the enzymatic medium and the NADPH generating system, to identify the activity of the diverse CYP450 isoenzymes and/or flavin-containing monooxygenases, which are all NADPH-dependent enzymes; a sample without the NADPH generating system, to identify the activity of carboxylesterases, which do not depend on NADPH to exert their activity; and a sample without the microsomal fraction, to assess the stability of compounds in the reaction medium.

##### Experimental Lipophilicity Determination via Distribution Coefficient (log­(D)_7.4_)

The analysis was performed using LC-MS/MS (liquid chromatography-tandem mass spectrometry). The chromatography system used was a Prominence UFLC (Shimadzu Corporation, Kyoto, Japan), interfaced with a LCMS-8045 triple quadrupole mass spectrometer (Shimadzu Corporation, Kyoto, Japan) equipped with an electrospray ionization source (ESI).

For the determination of the distribution coefficient (elog­(D)), a methodology based on the retention time of molecules in the stationary phase was used. The chromatogram was achieved on a Supelco Ascentis express RP amide HPLC column (5 cm x 2.1 mm, 2.7 μM), using 5% methanol in 10 mM ammonium acetate pH 7.4 (A) and 100% methanol (B) as mobile phases. The mobile phase was eluted in binary gradient mode, as follows: 0 min: 95% A; 0.3 min: 100% A; 5.2 min: 0% A; 5.6 min: 0% A; 5.8 min: 100% A; 7.0 min: 100% A. The total run time was 7 min, and 5 μL of each sample were injected. The solutions of tested compounds were prepared at a concentration of 1.0 mg/mL in compound by adding the stock solution to a (1:1) mix of mobile phases A:B (internal standard at 200 nM), and the DMSO concentration was lower than 2%. The lipophilicity of compounds was assessed by injecting individually the test compounds and a series of eight commercial drugs, covering a log­(D) range of −1.86 to 6.1 (acyclovir: −1.86, atenolol: 0.16, antipyrine: 0.38, fluconazole: 0.50, metoprolol: 1.88, ketoconazole: 3.83, tolnaftate: 5.40, and amiodarone: 6.10).^75–77^ The retention time (in minutes) of each of the eight standards was plotted against their log­(D) values, and the resulting equation for the calibration curve (y = mx + b) was used to calculate the elog­(D) values for the compounds from their retention time.

##### Kinetic Solubility

The analysis was performed using LC-MS/MS (liquid chromatography-tandem mass spectrometry). The LC-MS/MS system used consisted of a Prominence UFLC (Shimadzu Corporation, Kyoto, Japan) chromatography system, interfaced with a LCMS-8045 triple quadrupole mass spectrometer (Shimadzu Corporation, Kyoto, Japan) equipped with an electrospray ionization source (ESI).

To determine kinetic solubility, stock solutions of test compounds and controls (10 mM in DMSO) were transferred to two 96-well plates (incubation plate) in duplicates. PBS pH 7.4 or 2.0 (final concentration of 250 μM) was added to each well of the plates, and the DMSO concentration was kept lower than 2.5%. The plates were sealed and shaken for 24 h (200 rpm/25 °C). The precipitates on the incubation plate were removed by centrifugation (15 min/3000 rpm/25 °C), and the supernatant fractions were quantified by LC-MS/MS. An intermediate standard solution diluted at 0.5 mM in acetonitrile:water (1:1) was prepared from a 10 mM standard solution. A calibration curve was prepared for each of the test compounds and controls by diluting several times the intermediate standard solution to reach the desired concentrations of 50, 40, 20, 2, and 1 μM. The resulting equation for the calibration curve (y = mx + b) was used to calculate the experimental concentration values for the compounds. The chromatogram for analysis was achieved on a Supelco Ascentis express C18 column (3 cm x 2.1 mm, 5 μM), using water +0.05% formic acid (A) and acetonitrile +0.05% formic acid (B) as mobile phases. The mobile phase was eluted in binary gradient mode, and the gradient was as follows: 0 min: 98% A; 1.2 min: 2% A; 2.0 min: 2% A; re-equilibration time: 0.6 min, 98% A. The total run time was 2 min, and 5 μL of each sample were injected and eluted at a flow rate of 0.6 mL/min.

##### Hepatotoxicity

HepG2 cells were seeded at 2 × 10^4^ cells/mL in DMEM, the plates were incubated for 24 h (5% CO_2_, 37 °C) and the compounds and doxorubicin (used as a positive control) were added in serial dilutions. After 72 h incubation, 15 μL of MTS reagent (CellTiter96) was added to each well, and the plates were incubated for 4 h then read using a microplate absorbance reader at 490 nm.^78^ Growth inhibition was expressed as a percentage of the absorbance of the negative control wells. The CC_50_ values were automatically calculated from the dose–response curves fitted using the log of the inhibitor concentration vs the normalized response between 0 and 100% with a variable slope.

#### Mode of Action Assays

##### Flow Cytometric Analysis of Sub G0/G1 of the Cell Cycle

*L. infantum* promastigotes (MHOM/MA/67/ITMAP-263) at a concentration of 1 × 10^6^ parasites/mL were treated with the compounds for 24 h at concentrations of 0.5 x IC_50_, IC_50_ and 2 x IC_50_ (IC_50_ values determined against *L. infantum* promastigotes), while untreated parasites were used as a negative control. Then, the cells were collected, washed twice with 1 × PBS, and fixed in 70% ethanol at 4 °C for 24 h. The fixed parasites were then washed with 1 × PBS and the pellet was treated with 500 μL of RNase A (20 mg/mL) and incubated at 37 °C for 1 h. Subsequently, the parasites were stained with 10 μg/mL propidium iodide (PI) in the dark for 20 min. Cell cycle distribution, determining the percentage of cells in G0, G1, S, and G2/M phases, was analyzed using an Accuri C5 BD flow cytometer. The histograms presented are representative of results obtained from three independent experiments.^79^

##### FACS Analysis for Determination of Phosphatidylserine (PS) Externalization

An annexin V/7-amino-actinomycin D (7-AAD) apoptosis detection kit (BD Bioscience) was used for the detection of apoptotic or necrotic cell death in *L. infantum* promastigotes. After 72 h incubation with the compounds at concentrations of 0.5 x IC_50_, IC_50_ and 2 x IC_50_ (IC_50_ values determined against *L. infantum* promastigotes), *L. infantum* promastigotes (10^6^ cells/mL) were washed in phosphate-buffered saline (PBS) medium and centrifugated at 13000 rpm for 5 min, then stained with annexin V-PE/7-AAD for 15 min in the dark at 4 °C. Data was acquired using an Accuri C5 BD flow cytometer.^80^

##### Statistical Analysis

All data is presented as Mean ± SD (standard deviation). Each experiment was conducted independently in triplicates. Statistical analysis was performed using an unpaired Dunnett test to assess significant differences between two groups, *p* < 0.05 was set as a significant level.

##### Reactive Oxygen Species Production Assay

The fluorescent dye H_2_DCFDA was employed to evaluate ROS production. *L. infantum* promastigotes (MHOM/MA/67/ITMAP-263) at a concentration of 2 × 10^6^ parasites/mL were treated either with **2** at a concentration equal to its IC_50_ against *L. infantum* promastigotes or with 100 μM H_2_O_2_ for 24 h. After treatment, the parasites were harvested, washed three times with 1 × PBS, and incubated with 10 μM H_2_DCFDA dye in 500 μM of 1 × PBS in the dark at room temperature for 30 min. Subsequently, the cells were centrifugated, resuspended in ice-cold PBS and kept at 0 °C until reading on a SpectraMax Gemini XPS/EM microplate reader. The median fluorescence intensity (MIF) was measured on a population of at least ten thousand cells and the data was further analyzed using Excel 2021.^79^

#### Enzyme Inhibition Assays

##### *L. infantum* GSK-3A and GSK-3B

For IC_50_ measurements, compounds **1** – **6** were serially diluted in 100% DMSO (14-point, 3-fold serial dilution, and highest final assay concentration of 30 μM) and transferred (100 nL) to the assay plate using an automated liquid transferring system (CyBio FeliX, Analytik Jena GmbH). The following controls were included: protein in 1% DMSO (100% activity), 10 μM AZD-5438 (0% activity), and buffer without enzyme (blank).

To detect the progress of the enzymatic reaction, a commercially available TR-FRET-based assay (LANCE TR-FRET, PerkinElmer) was used. In the enzymatic step, purified protein kinase and the peptide substrate were incubated with compounds **1** – **6** for 30 min at 25 °C before the addition of ATP to start the reaction, which was run at 30 °C (2 h for GSK-3A or 1 h 15 min for GSK-3B). The final concentrations of each component of the assays were: 50 nM GSK-3A or 10 nM GSK-3B, Km of ATP (6.2 μM ATP for GSK3A and 13.0 μM for GSK3B), and 50 nM peptide (ULight-4E-BP1 substrate, PerkinElmer #TRF0128) in 1x buffer containing 50 mM HEPES (pH 7.5), 10 mM MgCl_2_, 1 mM EGTA, 0.01% Tween-20 and 2 mM DTT (reaction volume = 10 μL). For detection, the plate was incubated for 1 h at 25 °C after the addition of 6 mM EDTA (to stop the reaction) and 2 nM antibody (Europium-antiphospho-4E-BP1, PerkinElmer #TRF0216) in 1x detection buffer (PerkinElmer #CR97–100) (final reaction volume = 20 μL). All enzymatic reactions were performed in duplicates, and protein concentration was estimated using a NanoDrop2000 UV/vis spectrophotometer (Thermo Scientific), using the molecular weight and molar extinction coefficient of the protein.

The fluorescence signal was measured using a CLARIOstar microplate reader (BMG LABTECH) set with an excitation wavelength of 320 nm and an emission wavelength of 665 nm. MARS software (BMG LABTECH) was used to analyze the data obtained after reading each plate. The background fluorescence signal (blank) was subtracted from the fluorescence signal obtained for each reaction, in order to reflect the specific signal of enzymatic activity. The data was then analyzed with the GraphPad Prism v10 software, using the equation “log­(inhibitor) vs response - variable slope (four parameters) - Y = Bottom + (Top-Bottom)/(1 + 10̂((LogIC50-X)* HillSlope))” to obtain dose–response curves and determine IC_50_ values. The Z’-factor was calculated for each assay individually (values between 0.5 and 1.0 are considered excellent).

##### *L. braziliensis* DHODH

The inhibition assay was conducted under the following conditions: 50 mM Tris (pH 8.15), 150 mM KCl, 50 μM DHO, 60 μM DCIP, and 0.1% Triton X-100. **2** was assayed in triplicate at a concentration of 100 μM, using 5% (v/v) DMSO to prepare stock solutions. The reaction was initiated by adding the enzyme, reaching a final enzyme concentration of 165 nM. Enzymatic activity was then measured for 60 s at 610 nm using a microplate reader (SpectraMax Plus 384, Molecular Devices, San Jose, CA, USA) at 25 °C. The uninhibited reaction, containing 5% (v/v) DMSO, was used as a positive control (100% activity).

##### *L. braziliensis* Hsp83 and SOD Interaction Screening by Differential Scanning Fluorimetry (DSF)

The assays were performed using an Applied Biosystems 7500 Fast RT-PCR system (Applied Biosystems, Foster City, CA, USA), in triplicate, with 96-well PCR plates (BioRad, Hercules, CA, USA) sealed with transparent capping strips (BioRad, Hercules, CA, USA). The plates were centrifugated for 2 min at 2000 rpm at 25 °C. The temperature was then ramped up from 25 to 85 °C, increasing 1 °C per minute, while fluorescence signals were monitored using SYPRO Orange dye, with an excitation wavelength of 492 nm and an emission wavelength of 610 nm.

The DSF conditions used in this study have been previously described.^81,82^ Briefly, each well contained 10 μM *L. braziliensis* Hsp90 or 5 μM *L. braziliensis* SOD, 1 μL of 5X SYPRO Orange, buffer A (50 mM PBS, 100 mM NaCl, pH 7.0 for *L. braziliensis* SOD or 50 mM Tris-HCl, 100 mM KCl, pH 8.0 for *L. braziliensis* Hsp90), and either 1 μL DMSO (5% v/v, negative control) or **1 – 6** (diluted in DMSO). Triplicates were run for all experiments. Raw fluorescence data was collected using the Applied Biosystems 7500 Fast Software v2.0 and then exported to NAMI^83^ for Tm calculation via the first-derivative method. Differences in Tm values (ΔTm) were considered statistically significant when *p* < 0.05, as determined by the Kruskal–Wallis test followed by Dunn’s post-test for multiple comparisons, performed using GraphPad Prism 10.0 (GraphPad Software, San Diego, CA, USA, www.graphpad.com).

### *In Vivo* Biological Assays in BALB/c Mice

All animal experiments were conducted in accordance with the guidelines and regulations of the Sociedade Brasileira de Ciência em Animais de Laboratório (SBCAL) and were approved by the Ethical Committee for Animal Experimentation of the Oswaldo Cruz Foundation – FIOCRUZ, under protocol number P52/22.2 (license no. LW-7/23). The study complies with the ACS Ethical Guidelines for the Publication of Chemical Research. BALB/c mice were obtained from the Centro de Bioterismo of the IRR/Fiocruz Minas. Free access to a standard diet was allowed during the whole study, and tap water was supplied *ad libitum*.

#### Acute Toxicity In Vivo

The maximum tolerated dose (MTD) of **2** was determined in BALB/c mice. One male and one female specimen, uninfected, 6 weeks old (ideal weight: 20 g), were each administered intraperitoneally an emulsified sample prepared with 70% PBS1X, 20% PEG 400 (Polyethylene glycol 400, Sigma-Aldrich), 0.16% Tween 80 and 10% of **2**, with an initial dose of 5 mg/kg of **2**. After 2 h, an additional dose of 15 mg/kg was administered (resulting in an accumulated dose (AD) of 20 mg/kg). After another 2 h, a new dose of 30 mg/kg was administered (AD = 50 mg/kg). After a further 2 h, a final dose of 50 mg/kg (AD = 100 mg/kg) was administered. Twenty-four hours after the administration of the final dose, both mice were inspected for toxic and subtoxic clinical manifestations according to Organization for Economic Co-operation and Development (OECD) rules.^84,85^ The inspection revealed no apparent signs of acute toxicity in both specimens.

#### Antileishmanial Efficacy in BALB/c Mice

BALB/c mice (male, 6 to 8 weeks old, 18 to 20 g) were inoculated intravenously (tail vein) with 2 × 10^7^ late-log-phase *L. infantum* promastigotes from *Li::tdTomato* lines, and randomly assigned into one of the four treatment groups (six mice per group). After 7 days of infection, each group was treated accordingly for 10 consecutive days: the positive control group was administered intraperitoneally in a single dose 500 mg/kg/day meglumine antimoniate (Glucantime, Sanofi Medley), both groups treated with **2** received two daily intraperitoneal injections totaling either 50 mg/kg/day or 100 mg/kg/day of **2**, and the control group was administered the vehicle solution. For the intraperitoneal injection, **2** was formulated as follows: a stock solution of **2** in DMSO (100 mg/mL) was prepared daily, and a solution of 70% PBS1X, 20% PEG 400 (Polyethylene Glycol 400, Sigma-Aldrich), 0.16% Tween 80 and 10% of **2** (from the stock solution) was prepared. The control group was also administered this solution, replacing **2** with 10% DMSO. All mice were euthanized 3 days after the end of the treatment, and the liver and spleen of each animal were collected.

The number of viable parasites in each organ was determined using a quantitative limiting dilution assay. Briefly, the organs were macerated using an Ultra-Turrax disperser (IKA-Werke GmbH & Co. KG., Staufen, Germany), and a tissue homogenate was obtained with 1 mL of M199 medium. Each tissue homogenate was sequentially diluted (10-fold) in 96-well flat-bottom microtiter black plates and incubated at 26 °C for 10 days. The wells containing motile promastigotes were identified with an inverted microscope (Axiovert 25, Zeiss), and the parasite burden was determined from the highest dilution at which promastigotes had grown after 10 days of incubation.

### *In Silico* Target-Fishing

The chemical similarity principle-based target fishing approach used to screen the molecular targets of **2** was adapted from ref 86. Briefly, the Simplified Molecular Input Line Entry Specification (SMILES) of **2** was uploaded to the Superpred (https://prediction.charite.de/, accessed on 14 June 2023),^87^ SwissTargetPrediction (https://www.swisstargetprediction.ch/, accessed on 14 June 2023),^88^ Similarity Ensemble Approach (SEA) (https://sea.bkslab.org/, accessed on 14 June 2023),^89^ and PharmMapper (https://www.lilab-ecust.cn/pharmmapper/, accessed on 14 June 2023)^90^ servers. Threshold values were selected by default parameters. A Venn diagram with the predicted molecular targets found by each server was generated with the “Calculate and draw custom Venn diagrams” web tool (http://bioinformatics.psb.ugent.be/webtools/Venn/). Since the main means offered by the servers to evaluate target fishing are related to human proteins, a cross-reference with *L. infantum* (taxid: 5671) and *L. braziliensis* (taxid: 37617) related proteins was performed using the BLASTp program,^91^ applying filters set as an expected value (e-value) lower than 0.005 and a hit score larger than 100.0 for sequences to be considered homologous.^92^ Afterward, the resulting sequences were uploaded to the Cytoscape platform,^93^ where the plugin *“stringApp”* was used for the functional enrichment and picturing of the networks.^94^ Bubble plots were generated for visualization of the enrichment analysis within the SRplot online platform (http://www.bioinformatics.com.cn/SRplot).^95^ The *“rBLAST”* package was used for protein sequence comparison in the R programming environment (version 4.0.3), whereas the *“ClustalW”* option was used for the alignment of sequences, and the *“ggmsa”* package was used for visual representation.^96^ The FASTA format of the selected sequences were uploaded to the Iterative Threading Assembly Refinement (I-TASSER) server, where molecular models were predicted and obtained in PDB format.^97^ The ones with the highest confidence score (c-score) represented the best model. The active sites for each molecular model were identified by using the DoGSiteScorer server,^98^ while the molecular docking simulations were performed using the AutoDock Vina tool.^99^ The predicted molecular models were uploaded as macromolecules, and a thorough search was carried out by enabling the “Run AutoGrid” option, which creates configuration files for the grid parameter’s lowest energy pose, and then the “Run AutoDock” option, which uses the Lamarckian GA docking algorithm. The docking simulation was then run with an exhaustiveness setting of 20 and instructed to produce only the lowest energy pose.

## Supplementary Material



## Notes

NMR and HRMS spectra of compounds **1** – **6**, HPLC traces of **2** and **4 – 6**, and detailed results of MoA predictions can be found in the Supporting Information (PDF)

## ABBREVIATIONS

ADME: Absorption Distribution Metabolism and Excretion
Ar atm.: argon atmosphere
CC_50_: half maximal cytotoxic concentration
CYP450: cytochrome P450 enzymes
DCM: dichloromethane
DMF: *N,N*-dimethylformamide
EDC: 1-ethyl-3-(3-(dimethylamino)­propyl)­carbodiimide hydrochloride
eq: equivalent(s)
EtOAc: ethyl acetate
H-bond: hydrogen bond
Hex: hexane
HOBt: 1-hydroxybenzotriazole
IC_50_: half maximal inhibitory concentration
iPrOH: isopropanol
*L. braziliensis*: *Leishmania braziliensis*
*L. donovani*: *Leishmania donovani*
*L. infantum*: *Leishmania infantum*
LeishBox: library of hit compounds against *Leishmania*
MeOH: methanol
MLM: mouse liver microsomes
MTD: maximum tolerated dose
NTD: neglected tropical disease
PAINS: Pan-Assay Interference Compounds
PAMPA: Parallel Artificial Membrane Permeability Assay
rt: room temperature
SAR: structure–activity relationship
SI: selectivity index
THP-1: human leukemia monocytic cell line macrophages
VL: visceral leishmaniasis
