# Supplementary material for: Hit-to-Lead Studies of Pyrazinylpiperazines against Visceral Leishmaniasis: Pharmacokinetic Profile and In Vivo Efficacy of Potent Compounds against Leishmania infantum
Source: ACS Pharmacol Transl Sci. 2025 Jul 18;8(8):2736–55. doi: 10.1021/acsptsci.5c00318 (PMC12384620; doi:10.1021/acsptsci.5c00318)
Supplement: Supplementary file 1 [file pt5c00318_si_001.pdf]

Supporting Information for

Hit-to-lead studies of pyrazinylpiperazines against visceral leishmaniasis: Pharmacokinetic profile and *in vivo* efficacy of potent compounds against *Leishmania infantum*

Thibault Joseph William Jacques Dit Lapierre[‡a], Mariza Gabriela Faleiro de Moura Lodi Cruz[‡b], Gisele Barbosa[c], Analu R. Costa[d], Miguel Angel Chávez-Fumagalli[e], Thamires Quadros Froes[f, g, h], Priscila Zonzini Ramos[i], Paula Derksen Macruz[j], Thalita Carolyne Souza Trindade[f], Eduardo Jorge Pilau[j], Patricia Sampaio Tavares Veras[f], Maria Cristina Nonato[g,h], Katlin B. Massirer[i], Leonardo L. G. Ferreira[d], Adriano D. Andricopulo[d], Lídia Moreira Lima[c], Silvane Maria Fonseca Murta[b]*, Celso de Oliveira Rezende Júnior[a]*

[a] Laboratório de Síntese de Candidatos a Fármacos, Instituto de Química, Universidade Federal de Uberlândia (UFU), Uberlândia-MG, 38400-902, Brazil;

[b] Grupo de Genômica Funcional de Parasitos, Instituto René Rachou, Fundação Oswaldo Cruz (FIOCRUZ Minas), Belo Horizonte-MG, 30190-002, Brazil;

[c] Laboratório de Avaliação e Síntese de Substâncias Bioativas (LASSBio ®), Universidade Federal do Rio de Janeiro, Rio de Janeiro 21941-902, RJ, Brazil;

[d] Laboratório de Química Medicinal e Computacional (LQMC), Instituto de Física de São Carlos (IFSC), Universidade de São Paulo (USP), São Carlos-SP, 13563-120, Brazil;

[e] Computational Biology and Chemistry Research Group, Vicerrectorado de Investigación, Universidad Católica de Santa María, 04000 Arequipa, Peru;

[f] Laboratory of Host-Parasite Interaction and Epidemiology Gonçalo Moniz Institute - Fiocruz – Bahia, Salvador-BA, 40296-710, Brazil;

[g] Center for the Research and Advancement in Fragments and molecular Targets (CRAFT), School of Pharmaceutical Sciences at Ribeirao Preto, University of São Paulo, Ribeirão Preto 14040-903, SP, Brazil;

[h] Protein Crystallography Laboratory, Department of Biomolecular Sciences, School of Pharmaceutical Sciences at Ribeirao Preto, University of São Paulo, Ribeirão Preto 14040-903, SP, Brazil;

[i] Centro de Química Medicinal (CQMED), Centro de Biologia Molecular e Engenharia Genética (CBMEG), Universidade Estadual de Campinas (UNICAMP), Campinas, SP, 13083-886, Brazil.

[j] Laboratório de Biomoléculas e Espectrometria de Massas (LaBioMass), Universidade Estadual de Maringá (UEM), Maringá, PR 807020-900, Brazil.

[‡] These authors contributed equally.

[*] Corresponding authors: celso@ufu.br and silvane.murta@fiocruz.br

Table of contents

1. Detailed *in silico* mechanism of action (MoA) analysis of 2 .... S-4

2. NMR spectra of compounds 1 – 6 .... S-5

3. HRMS spectra of compounds 1 – 6 .... S-11

4. HPLC traces S-13

5. *L. braziliensis* Hsp83 and SOD inhibition assays S-15

6. *L. infantum* GSK-3A and GSK-3B inhibition assays S-16

1. Detailed *in silico* mechanism of action (MoA) analysis of 2

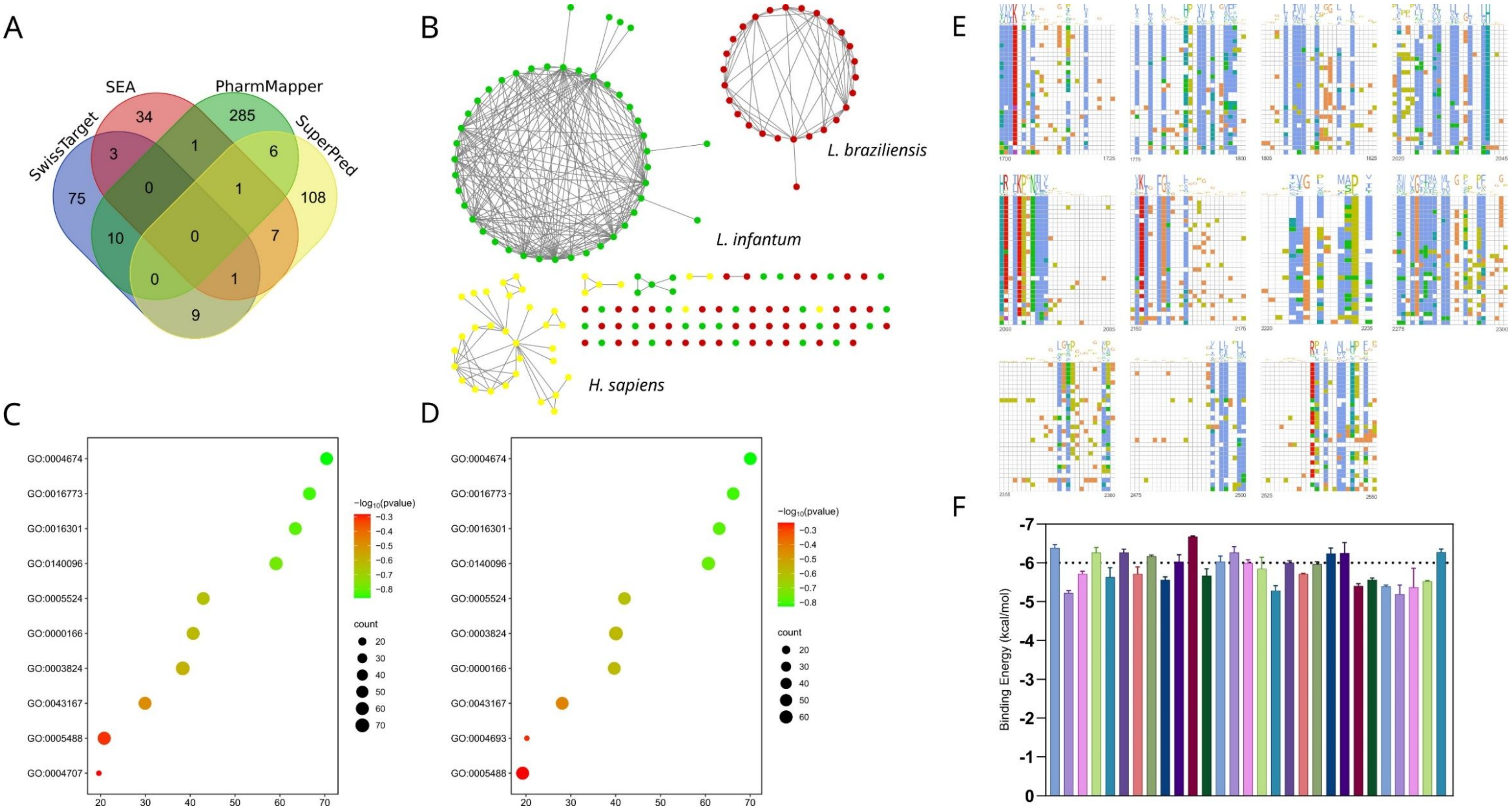

Figure S1. *In silico* target fishing prediction of the molecular targets of **2**. Venn diagrams of the number of overlapping "hits" predicted by online servers (A). Network diagram of proteins predicted to target **2** in *Homo sapiens* (yellow nodes), *L. infantum* (green nodes), and *Leishmania braziliensis* (red nodes) (B). Functional enrichment analysis on *L. infantum* (C) and *Leishmania braziliensis* (D) predicted molecular targets are shown in bubble plots. Sequence alignments display the conservation of residues for the selected sequences, while the x-axis represents the column numbers from the multiple sequences alignment (E). Binding affinity calculations for the selected molecular targets are shown in bar plots (F), while the dotted line represents the cut-off value (>6 kcal/mol). The 38 overlapping targets for more than two servers are: P11802, P14555, Q99884, P41594, O95271, P08473, P11309, P14780, P18031, P00533, P10828, P00918, P23946, P15121, P08254, P04150, Q9UKV0, P21397, O14672, P14867, P19634, Q969S8, Q9BY41, Q96DB2, Q00796, P09874, Q9H3R0, P29375, P43490, Q9Y5N1, P46098, Q99572, O75469, P43235, Q04609, P25774, P09211, P24941.

2. NMR spectra of compounds 1 – 6

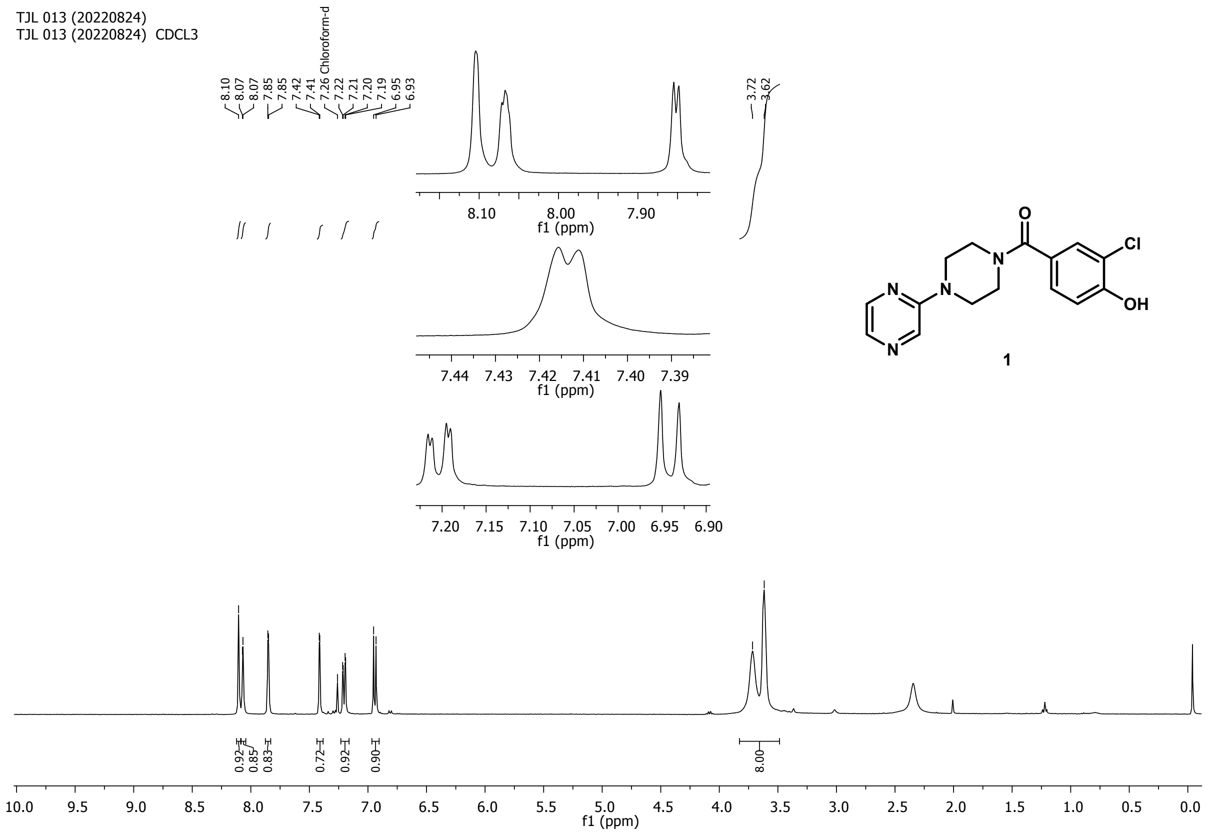

Figure S2. ^{1}H NMR spectrum of **1** (400 MHz, $CDCl_3$)

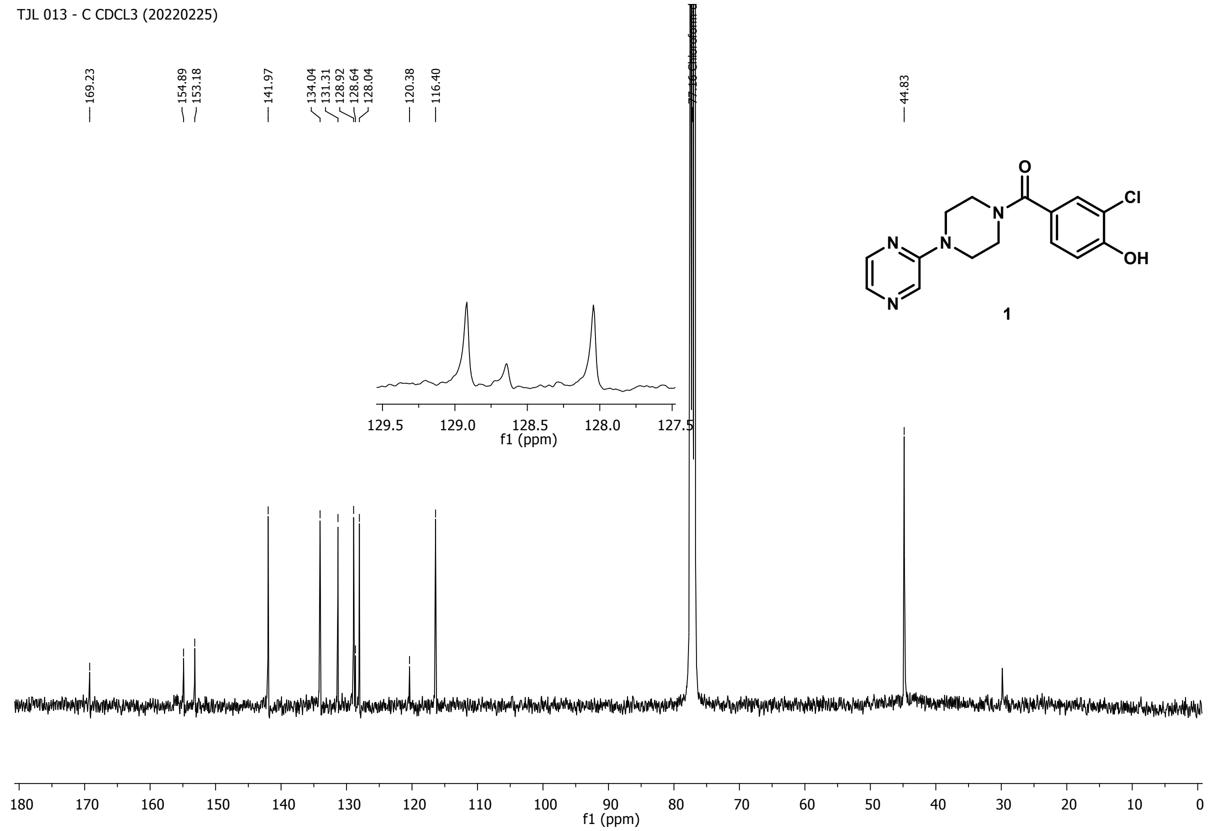

Figure S3. ^{13}C NMR spectrum of **1** (101 MHz, $CDCl_3$)

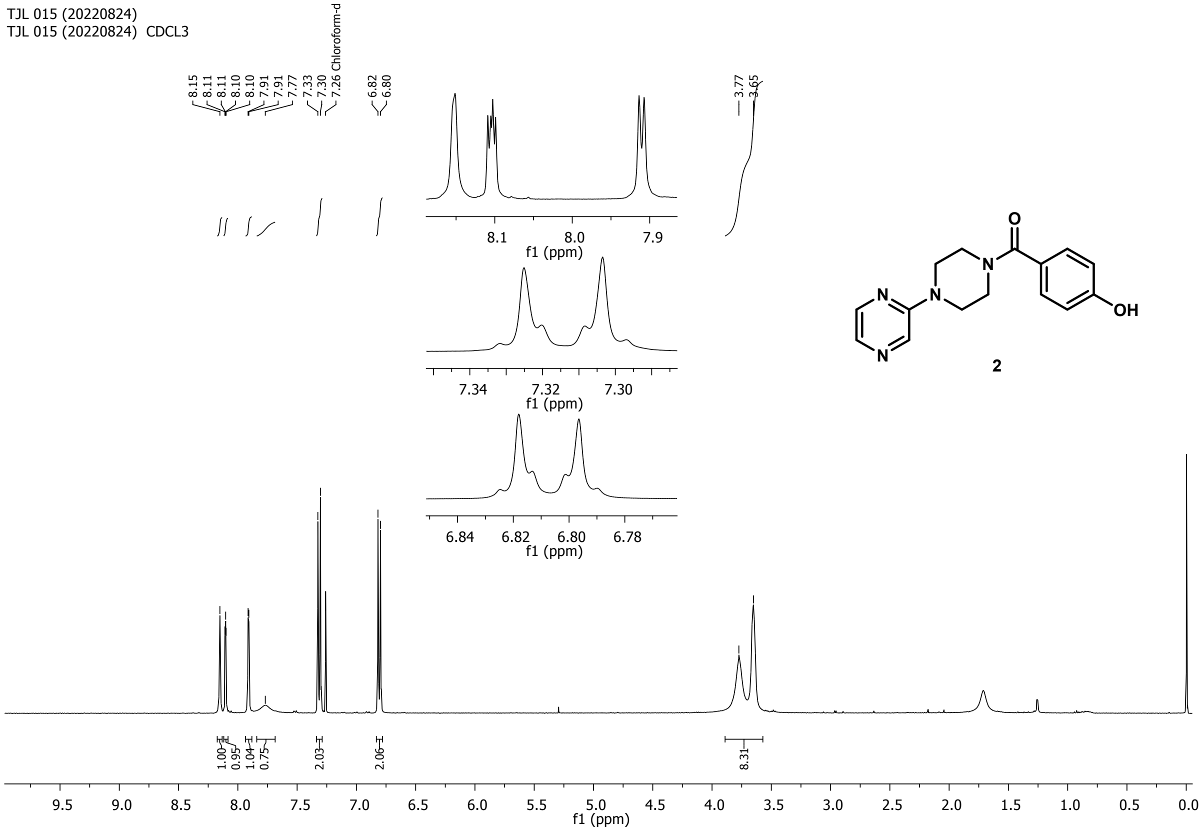

Figure S4. ^{1}H NMR spectrum of **2** (400 MHz, $CDCl_3$)

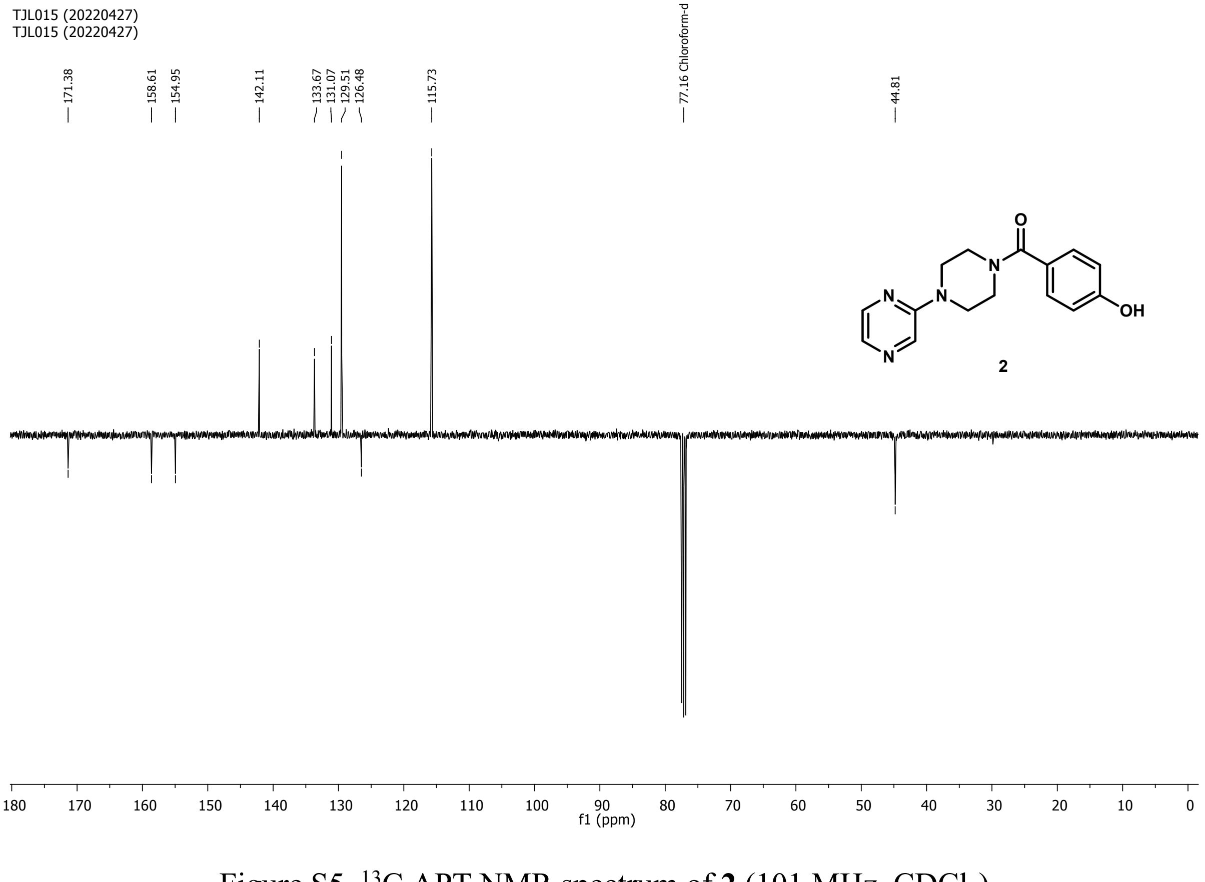

Figure S5. ^{13}C APT NMR spectrum of **2** (101 MHz, $CDCl_3$)

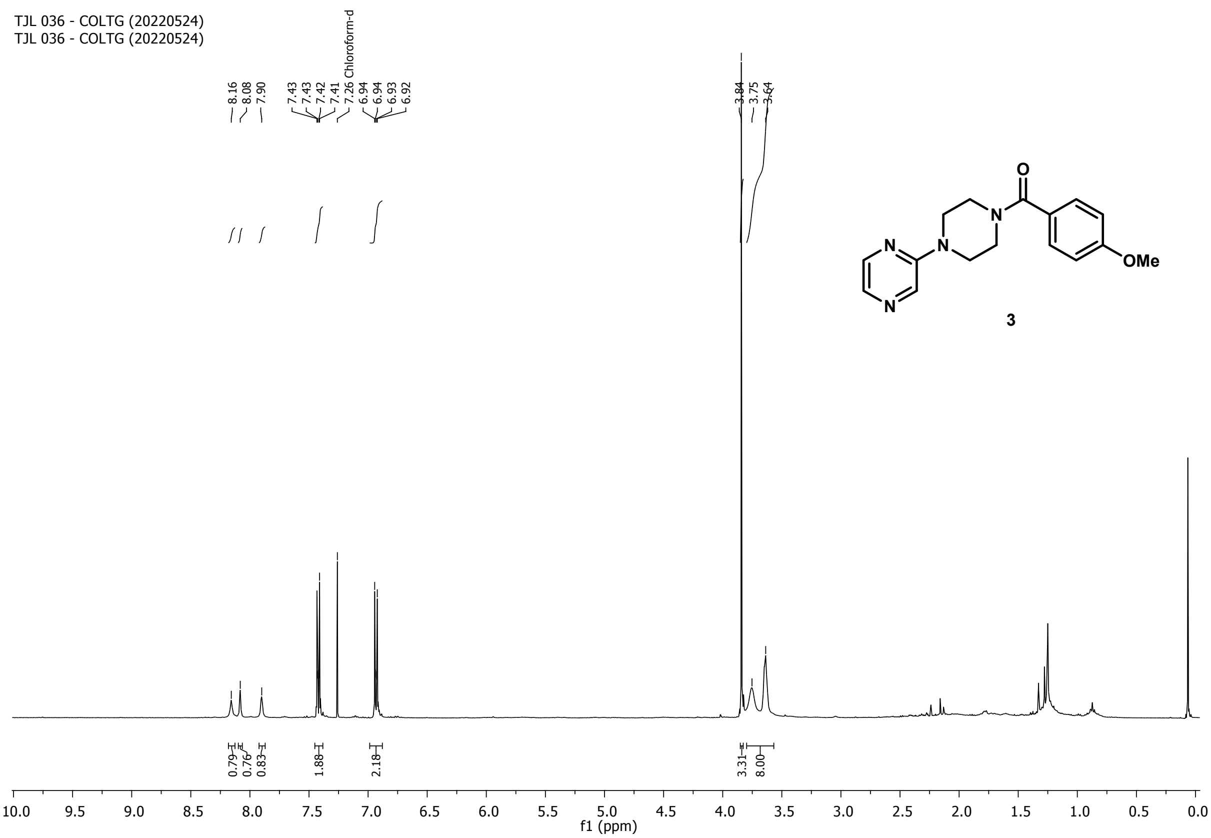

Figure S6. ^{1}H NMR spectrum of **3** (400 MHz, $CDCl_3$)

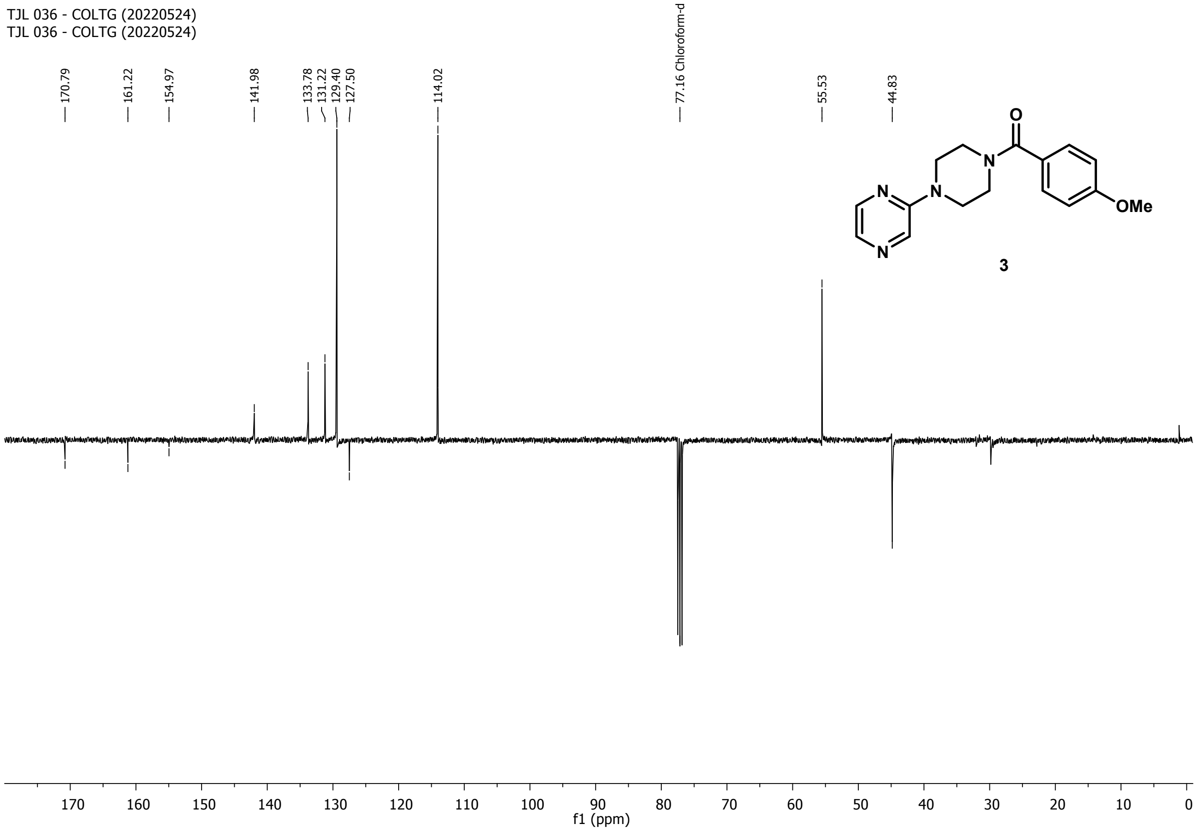

Figure S7. ^{13}C APT NMR spectrum of **3** (101 MHz, $CDCl_3$)

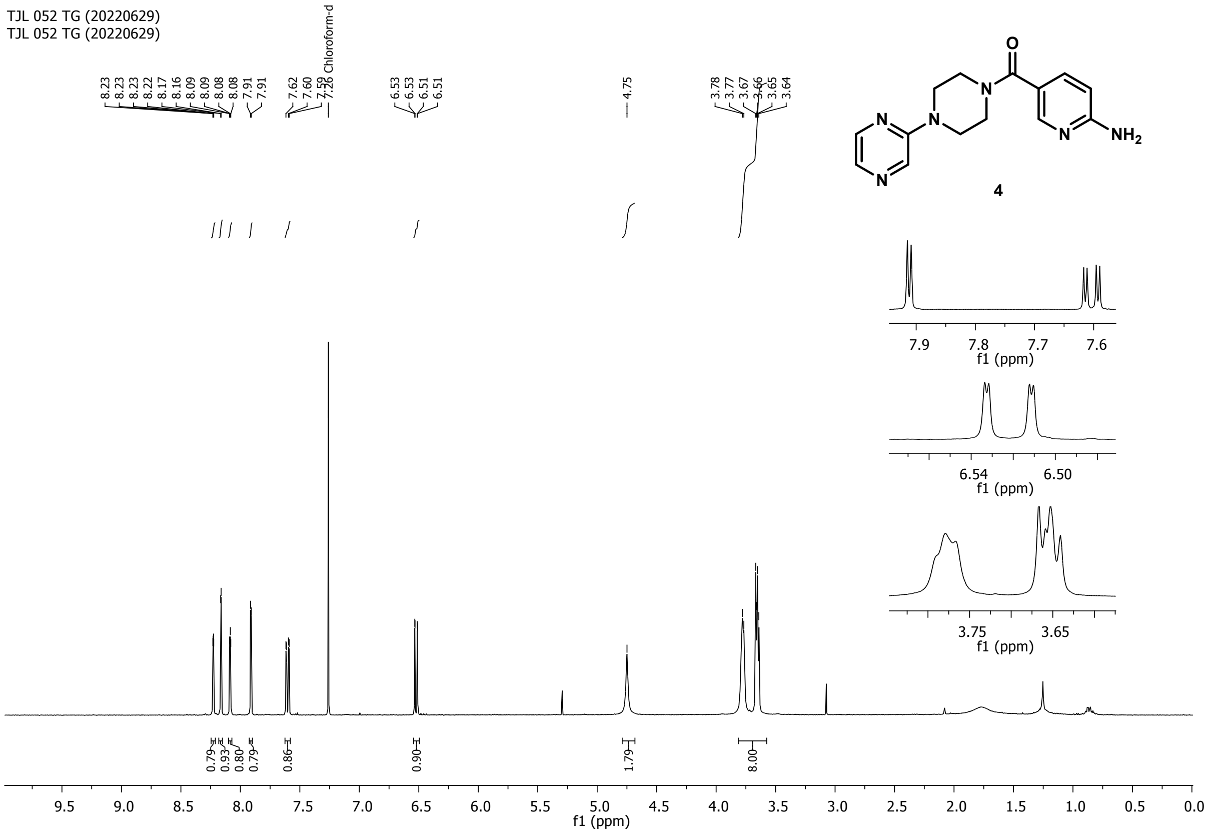

Figure S8. ^{1}H NMR spectrum of **4** (400 MHz, $CDCl_3$)

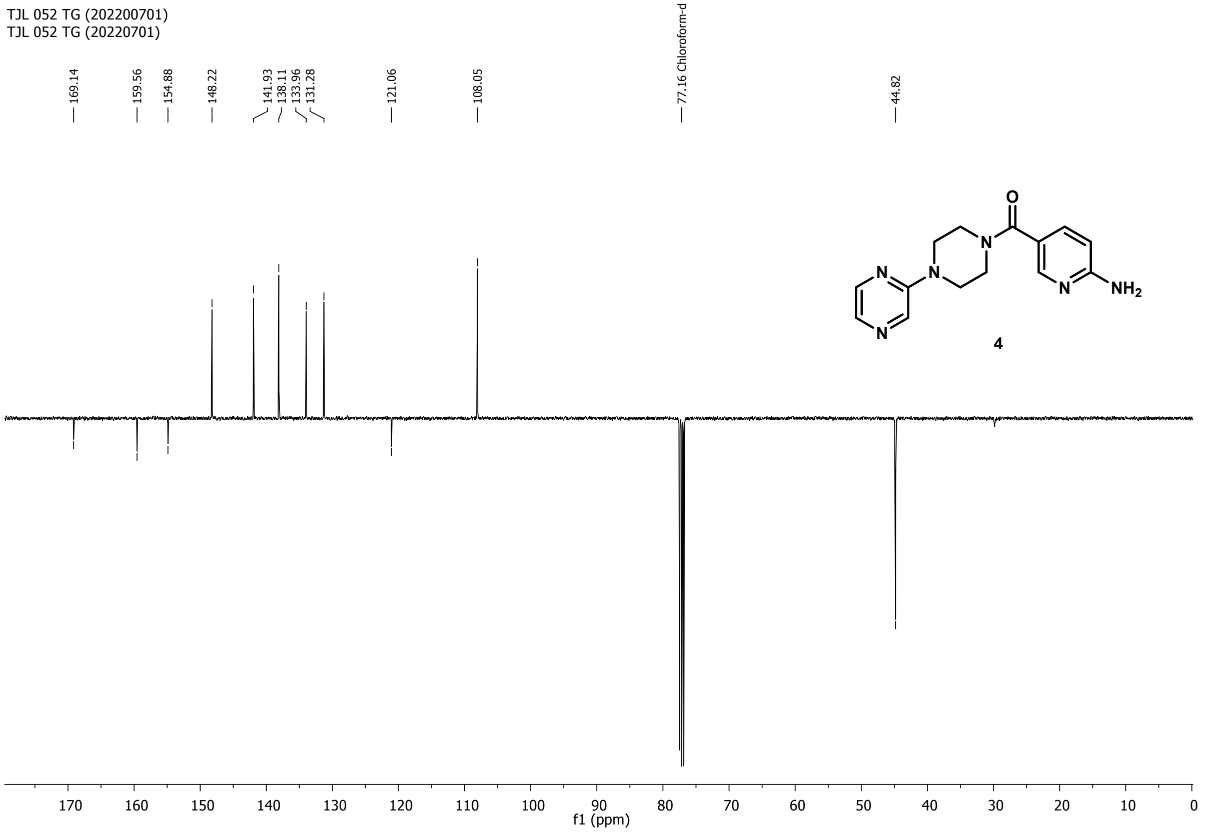

Figure S9. ^{13}C APT NMR spectrum of **4** (101 MHz, $CDCl_3$)

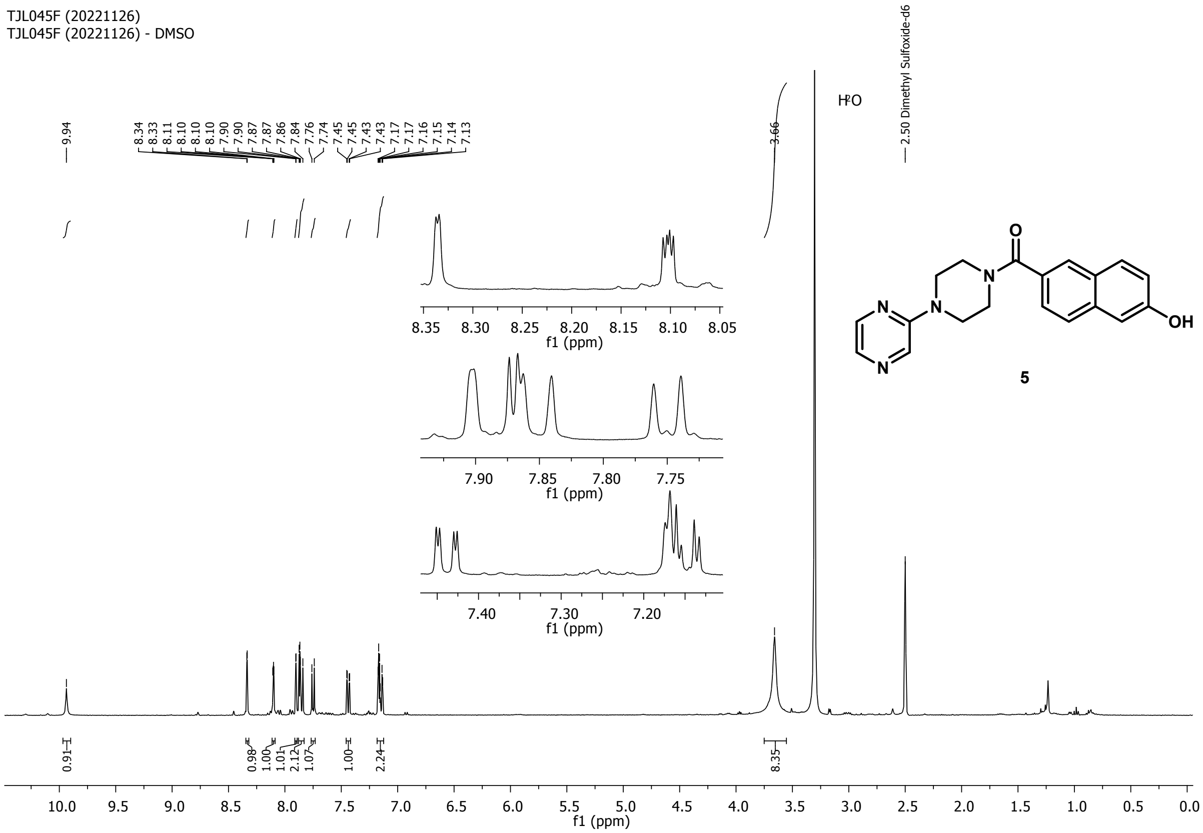

Figure S10. ^{1}H NMR spectrum of **5** (400 MHz, DMSO-d_6)

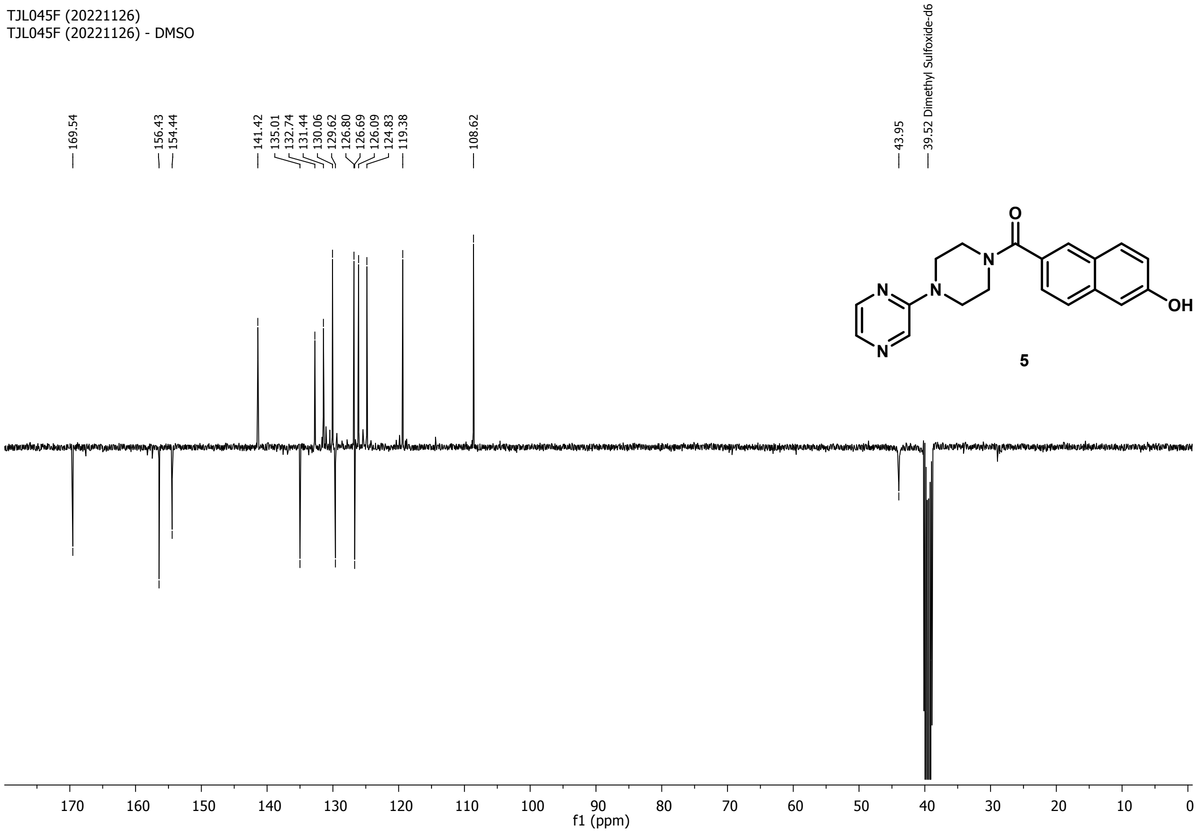

Figure S11. ^{13}C APT NMR spectrum of **5** (101 MHz, DMSO-d_6)

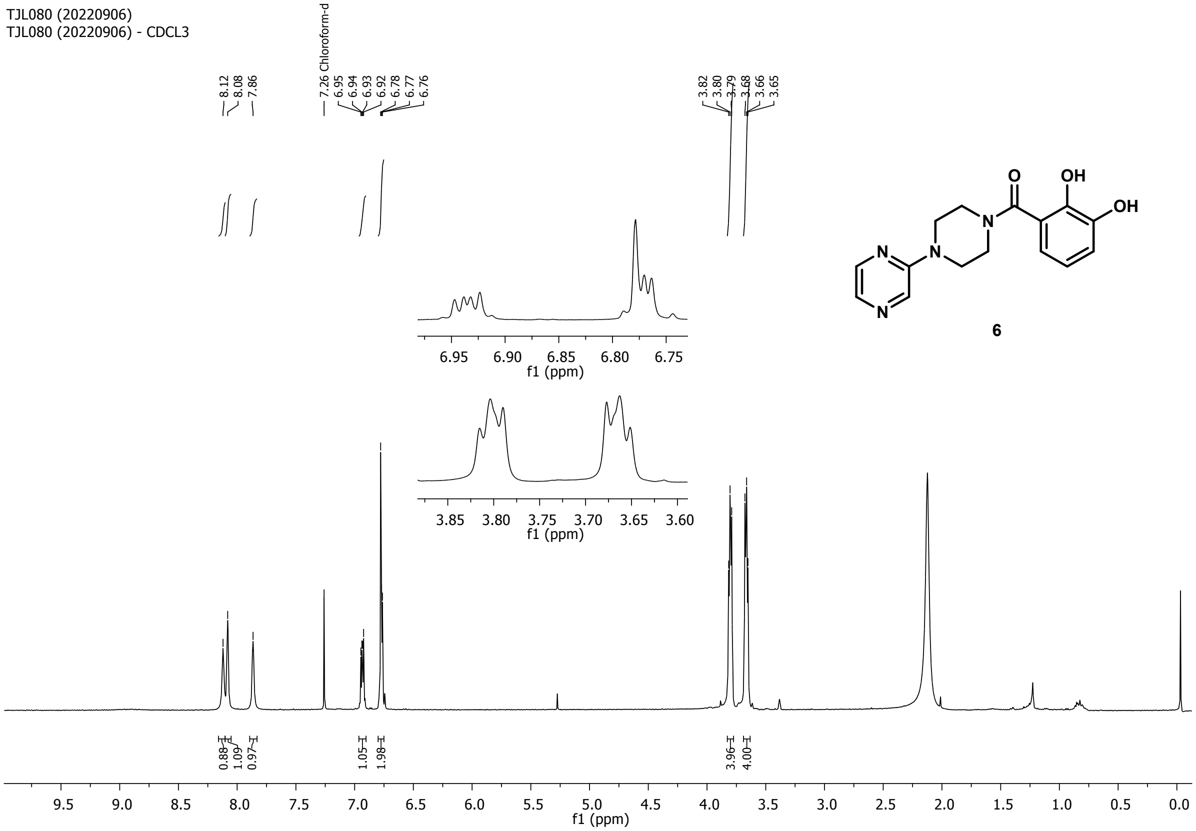

Figure S12. ^{1}H NMR spectrum of **6** (400 MHz, $CDCl_3$)

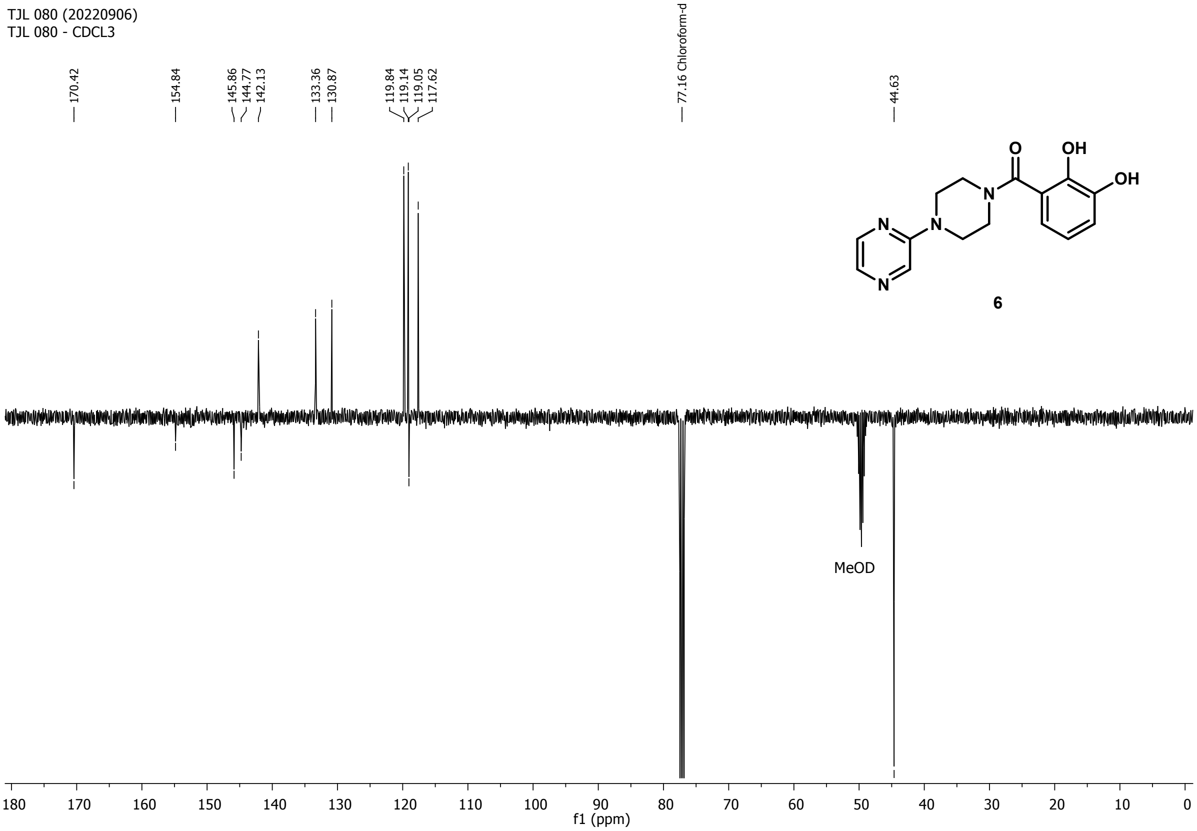

Figure S13. ^{13}C APT NMR spectrum of **6** (101 MHz, $CDCl_3$)

3. HRMS spectra of compounds 1 – 6

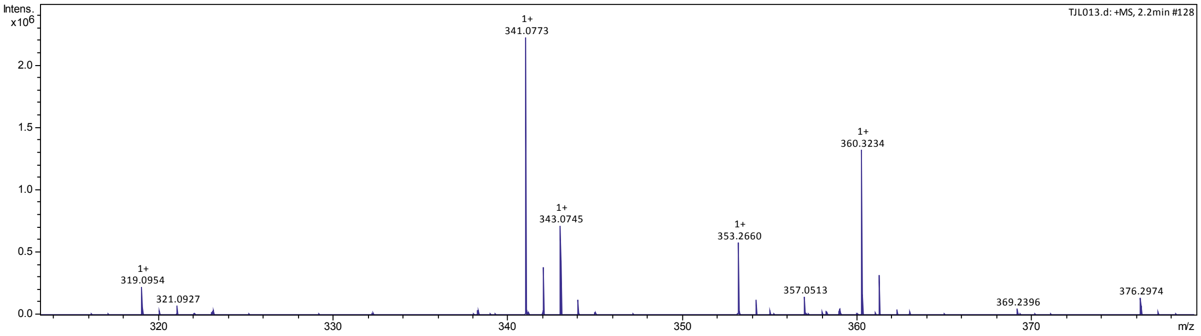

Figure S14. HRMS (ESI +) spectrum of **1**

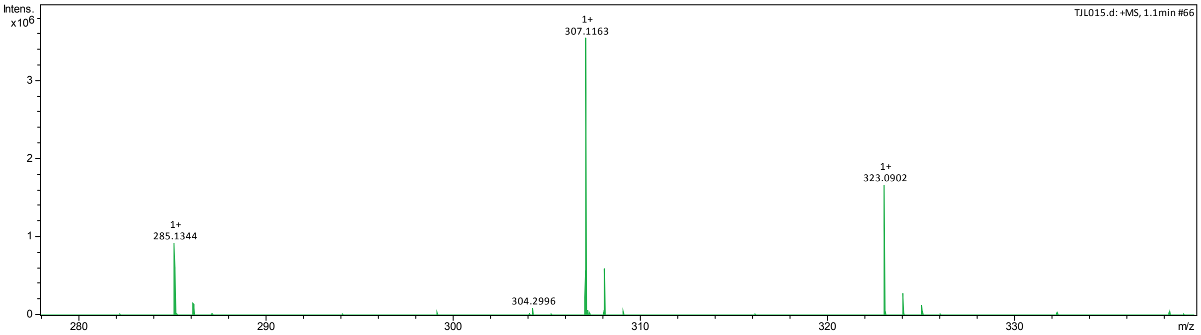

Figure S15. HRMS (ESI +) spectrum of **2**

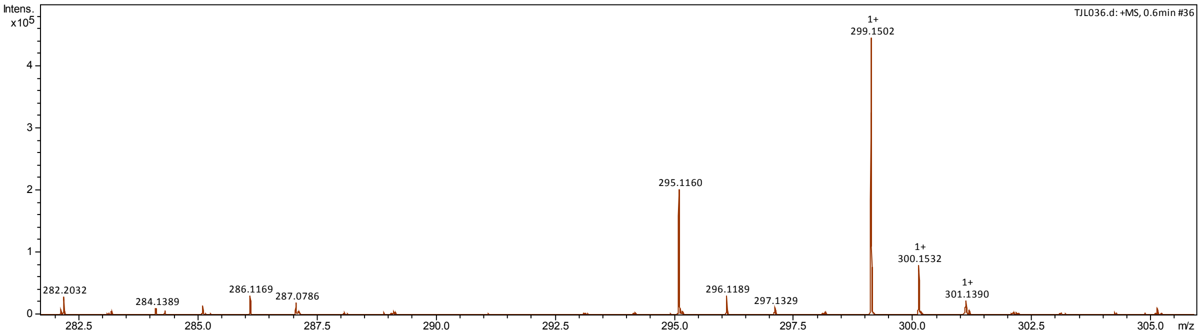

Figure S16. HRMS (ESI +) spectrum of **3**

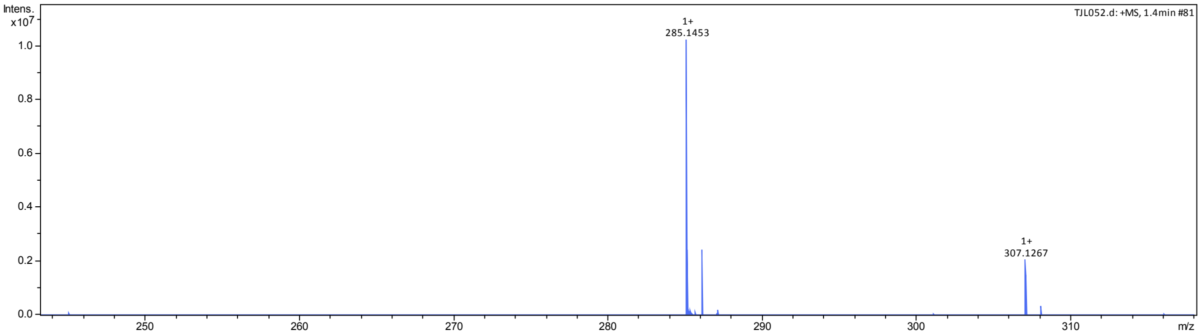

Figure S17. HRMS (ESI +) spectrum of **4**

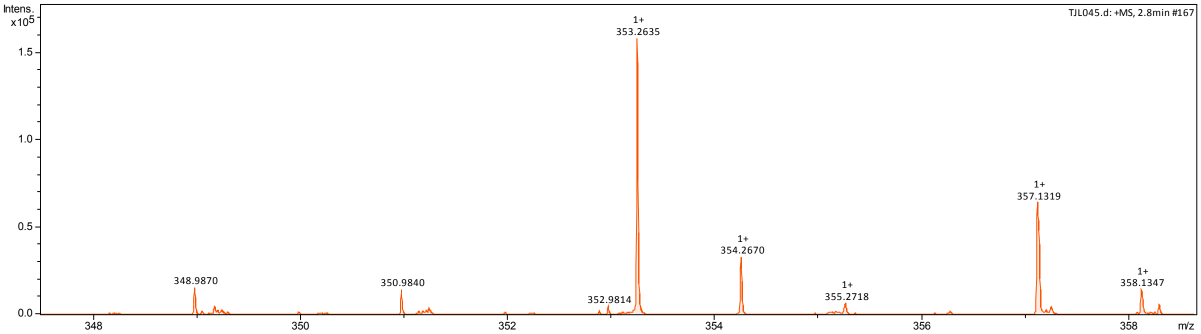

Figure S18. HRMS (ESI +) spectrum of **5**

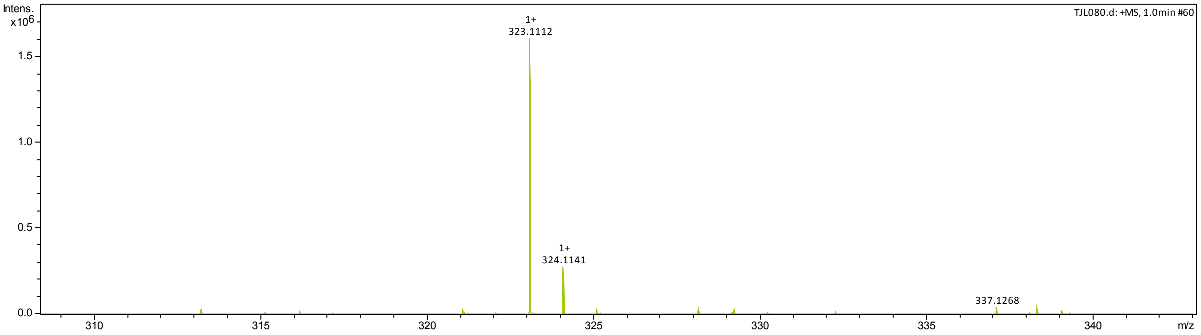

Figure S19. HRMS (ESI +) spectrum of **6**

4. HPLC traces

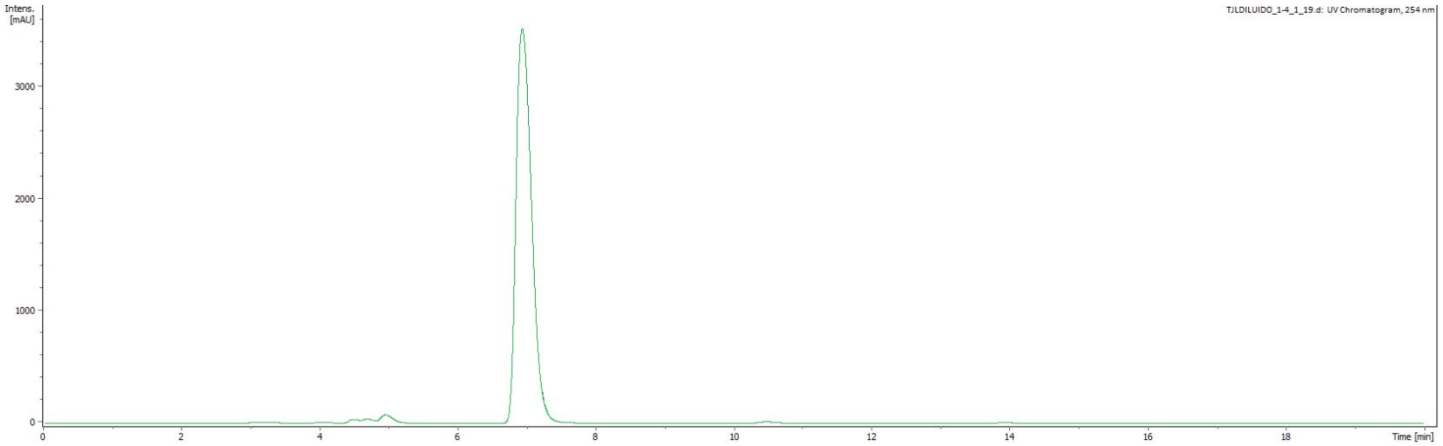

Figure S20. HPLC traces of **2**

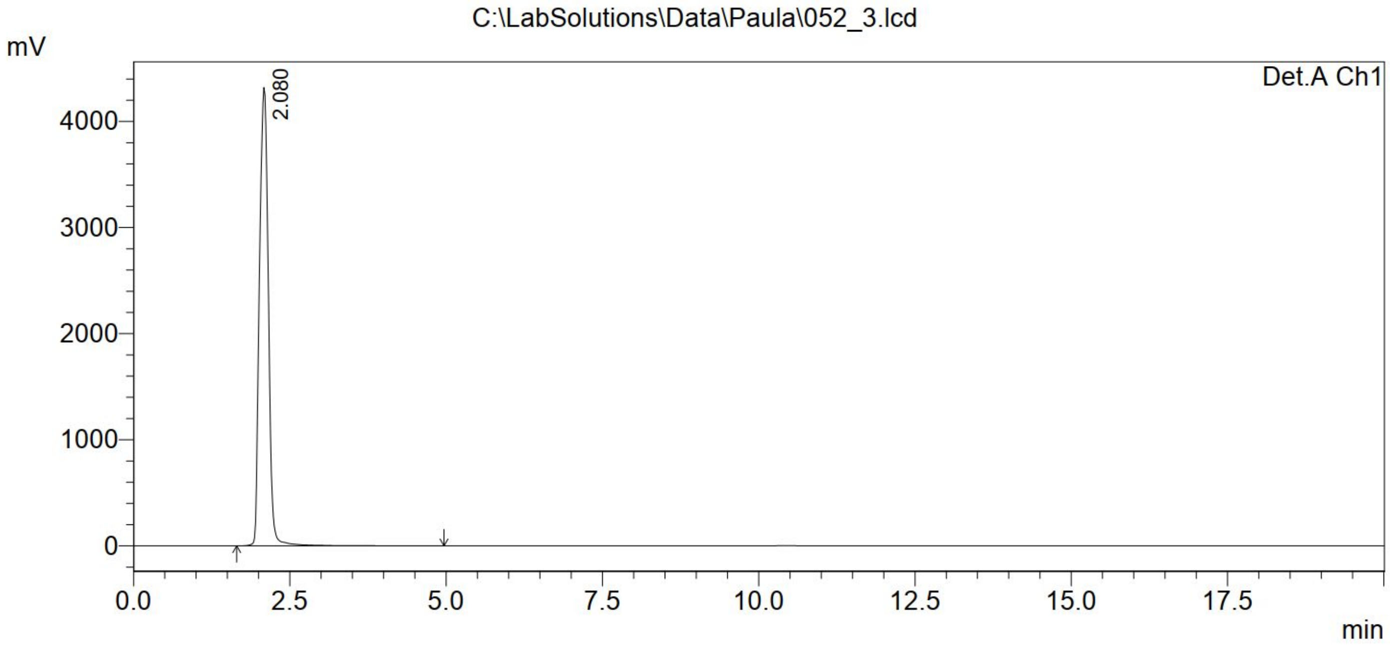

Figure S21. HPLC traces of **4**

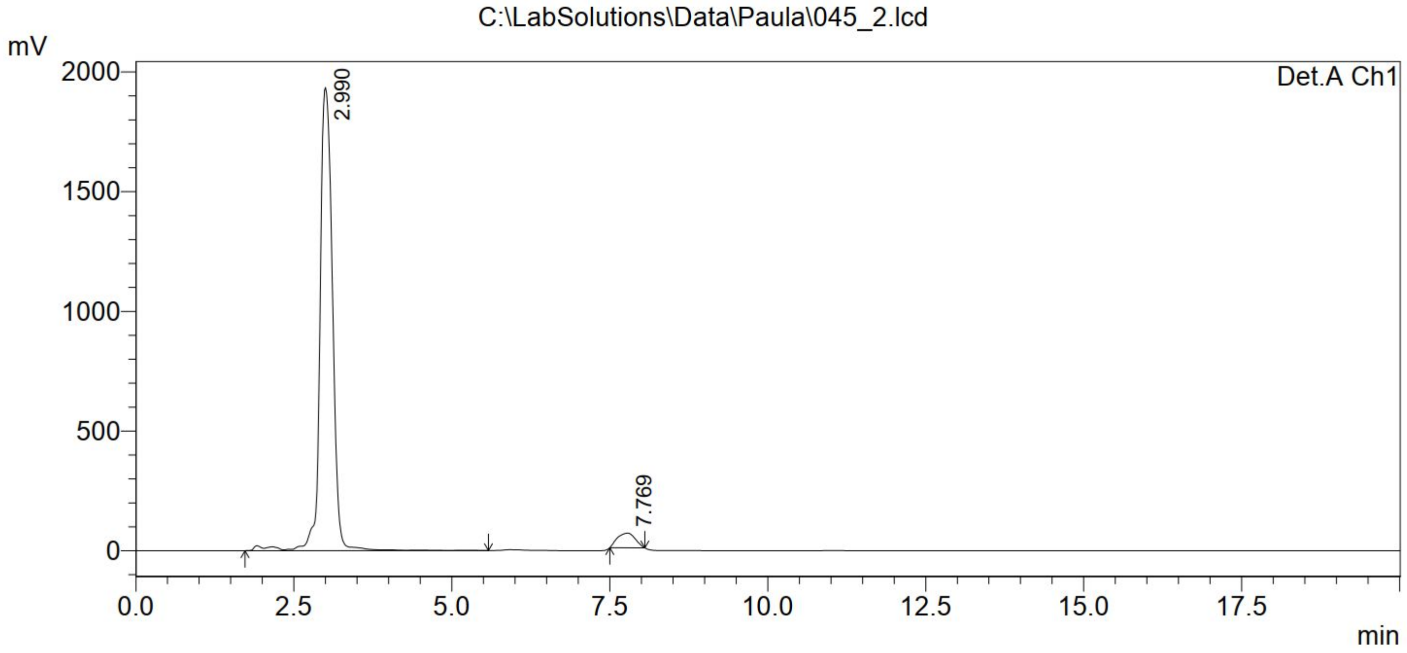

Figure S22. HPLC traces of **5**

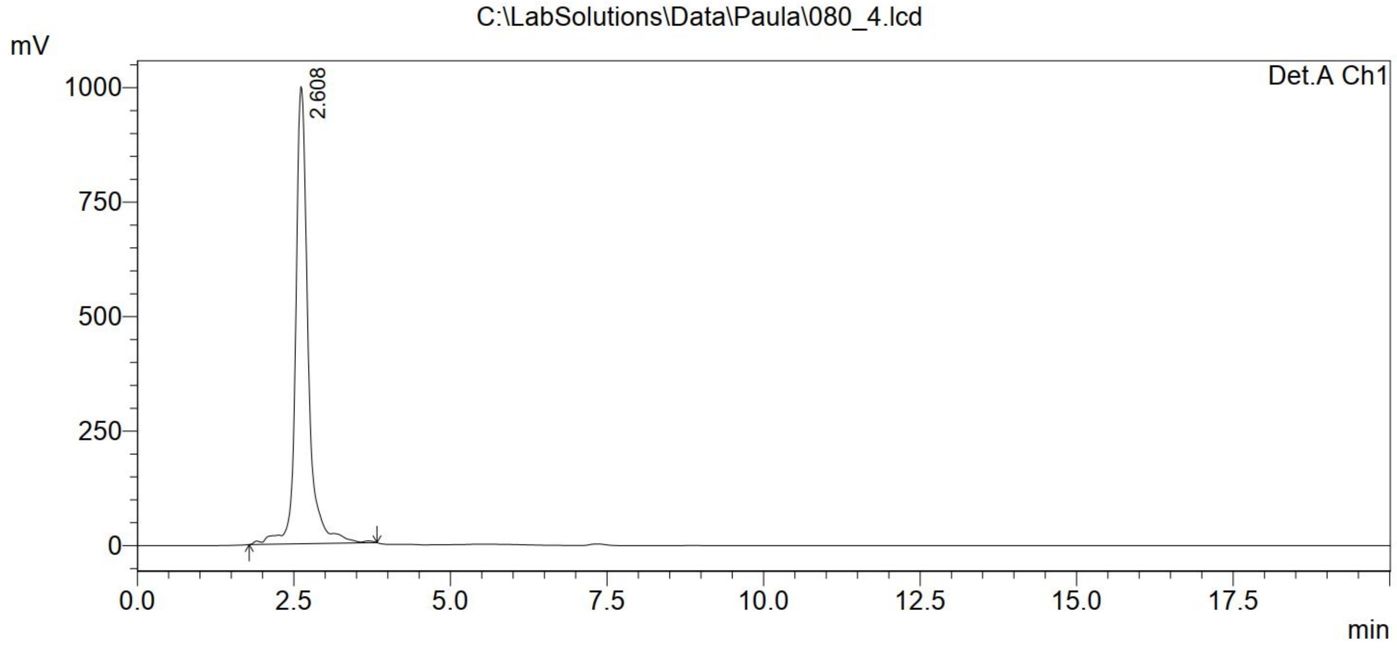

Figure S23. HPLC traces of **6**

5. *L. braziliensis* Hsp83 and SOD inhibition assays

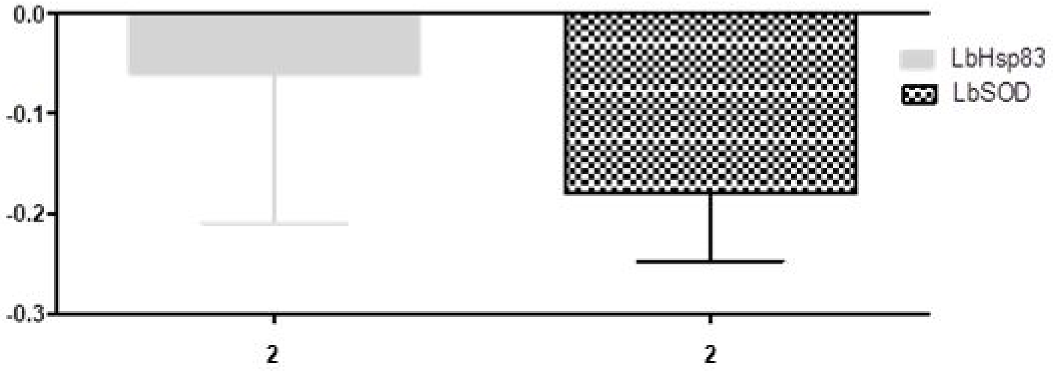

Figure S24. Inhibition of *Lb*Hsp83 and *Lb*SOD by compound **2** (100 µM)

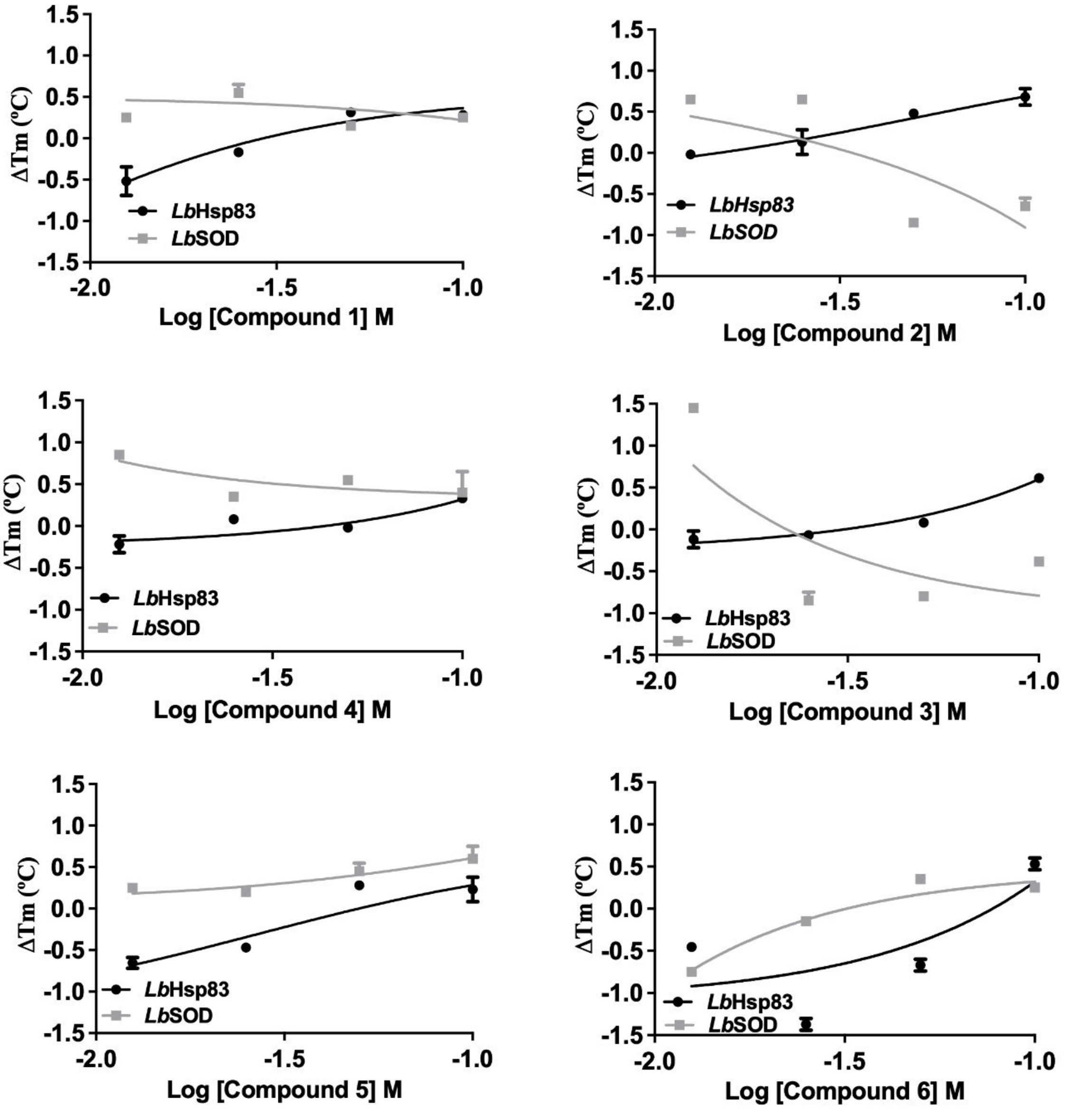

Figure S25. Titration curves for the inhibition of *Lb*Hsp83 and *Lb*SOD by compounds **1** – **6**

6. *L. infantum* GSK-3A and GSK-3B inhibition assays

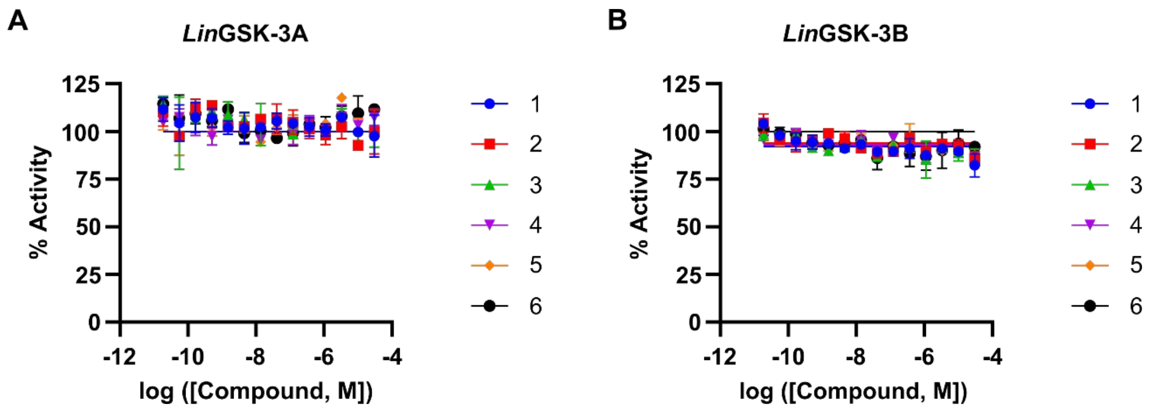

Figure S26. Enzyme activity assays after incubation of compounds **1** – **6** with *L. infantum* GSK-3A (A) and GSK-3B (B). Values are shown as percentual enzymatic activity.
